# A biomechanics and energetics dataset of neurotypical adults walking with and without kinematic constraints

**DOI:** 10.1038/s41597-024-03444-4

**Published:** 2024-06-18

**Authors:** Tomislav Baček, Mingrui Sun, Hengchang Liu, Zhongxiang Chen, Chris Manzie, Etienne Burdet, Dana Kulić, Denny Oetomo, Ying Tan

**Affiliations:** 1 The University of Melbourne, Department of Mechanical Engineering, 3010 Melbourne, Australia; 2 Monash University, Faculty of Engineering, 3800 Melbourne, Australia; 3 The University of Melbourne, Department of Electrical and Electronic Engineering, 3010 Melbourne, Australia; 4 Imperial College London, Department of Bioengineering, London, United Kingdom

**Keywords:** Databases, Musculoskeletal system, Biomedical engineering

## Abstract

Numerous studies have explored the biomechanics and energetics of human walking, offering valuable insights into how we walk. However, prior studies focused on changing external factors (e.g., walking speed) and examined group averages and trends rather than individual adaptations in the presence of internal constraints (e.g., injury-related muscle weakness). To address this gap, this paper presents an open dataset of human walking biomechanics and energetics collected from 21 neurotypical young adults. To investigate the effects of internal constraints (reduced joint range of motion), the participants are both the *control* group (free walking) and the *intervention* group (constrained walking - left knee fully extended using a passive orthosis). Each subject walked on a dual-belt treadmill at three speeds (0.4, 0.8, and 1.1 m/s) and five step frequencies ( − 10% to 20% of their preferred frequency) for a total of 30 test conditions. The dataset includes raw and segmented data featuring ground reaction forces, joint motion, muscle activity, and metabolic data. Additionally, a sample code is provided for basic data manipulation and visualisation.

## Background & Summary

Gait analysis is an essential tool for understanding human walking. Previous studies have demonstrated that humans optimise their gait, but the optimisation criteria may vary with individual health status and environmental conditions. Neurotypical adults tend to walk efficiently^1–3^ as predicted by simple pendulum models^4–7^. This is a consequence of the adaptations they make when walking at different speeds^8–13^, in the presence of changing external conditions^14–17^, and as they age^13,18–23^. Similar can be seen in people with physical impairments as they also adapt their gait^24–29^ albeit for different reasons than neurotypical individuals^30–34^. However, little is known about human adaptations on an individual level for several reasons. First, human studies are resource-intensive to conduct, making access to much-needed data severely limited (particularly for neurologically-impaired patients). Furthermore, the findings cited above come from studies designed-for-purpose and not publicly shared. Second, studies seldom investigate the effects of constraining normally available gait mechanisms (*internal* constraints; e.g., reducing muscle strength). One recent study that did^35^ has a small sample size and a small number of walking conditions tested (in terms of gait speed and gait cycles), limiting the analysis and outputs. Instead, these studies focus on imposing *external* constraints (e.g., walking speed^36,37^ and spatiotemporal parameters^3,38^). This is also the case with publicly available datasets^9,10,39–44^. Third, sex, age, and/or speed-matching, common in comparing gait across diverse cohorts^37,45^, is limited to group averages and trend comparisons. Consequently, an understanding of person-specific adaptations coming from individual biomechanical features is lacking.

Our study was designed to address these gaps and provide insights into individual gait adaptations when walking with *internal* constraints. A group of 21 neurotypical young adults (30 ± 8 years) was recruited for the study conducted in the CAREN biomechanics lab (Motek Medical B.V., the Netherlands) at The University of Melbourne, Australia. Neurotypical participants were chosen instead of neurologically impaired for two reasons. First, the demands of the study are too large for a patient group, and it would be unethical to impose further constraints on their restrained physical ability. Second, similar trends in neurotypical and impaired gait^24,25,33,34^ provide evidence that the underlying neuromusculoskeletal mechanisms governing human walking do not get overridden but simply change their manifestation in the presence of neurologically originated physical impairment. This suggests that insights gained from studying neurotypical populations could potentially be used to better understand patient walking as well, provided a cautious and informed approach is taken in translating the findings.

**Fig. 1:**
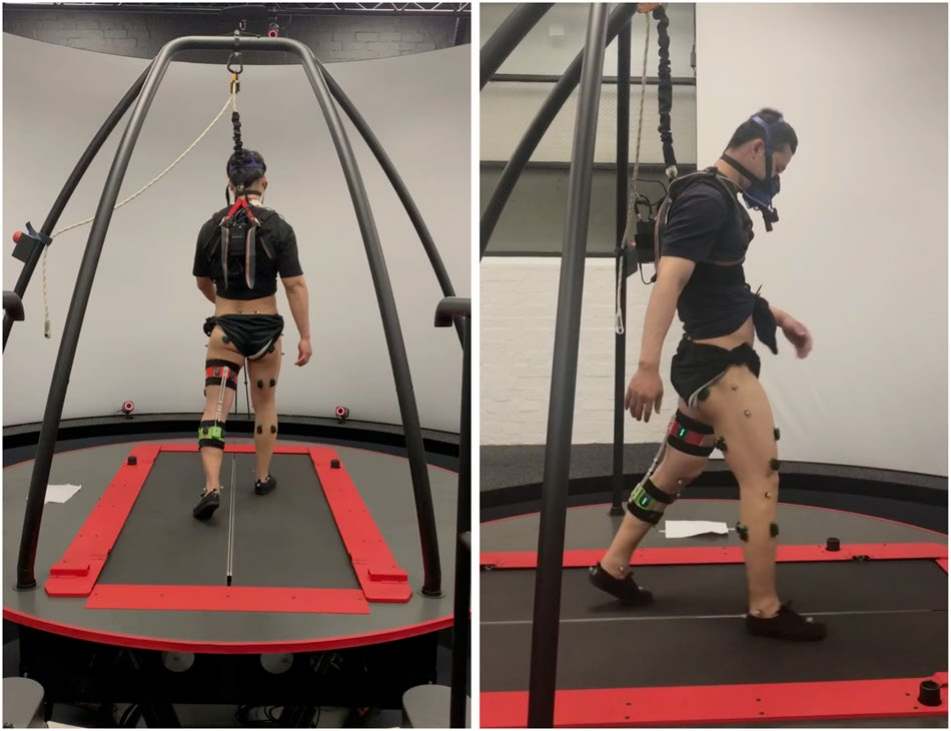
Experimental setup in the Motek CAREN biomechanics lab at The University of Melbourne, Australia. Participants walked on a dual-belt instrumented treadmill secured with an overhead harness and equipped with surface EMG sensors for measuring EMG amplitude (an estimate of muscle activity) on their lower limbs, retroreflective markers, an indirect calorimetry mask with an accompanying backpack, and, in half of the test conditions, a passive knee joint orthosis.

To allow the translation of the study findings into a better understanding of patient walking, the study was designed in discussion with experienced physiotherapists from two clinics in Melbourne. As a result, participants walked at two speeds relevant to hemiparetic patients (0.4 and 0.8 m/s) and one common in the neurotypical population (1.1 m/s). Preliminary tests showed that locking the knee joint using passive orthosis is most likely to simulate *hemiparetic conditions* and produce hip hiking (*‘unaffected frontal hip/pelvic angle when the affected limb is in mid-swing,’*^46^), circumduction (*‘greater-than-normal frontal thigh angle during mid-swing of the affected leg,’*^46^), and step-to-walking (a gait where *‘toe of the affected side does not pass the toe of the non-affected side in the stance phase’*,^47^), all common compensations in hemiparetic patients. This was demonstrated in our preliminary study in two participants^48^. The participants walked at five step frequencies (from − 10% to +20% of their preferred cadence) per speed since changing step length and time impacts both neurotypical and patient walking. The protocol was spread across three days/sessions to minimise fatigue and make the study technically and resource-wise feasible. Finally, to avoid problems of comparing data across different study designs, four different types of data relevant to characterise human walking was collected: ground reaction forces through a dual-belt instrumented treadmill, joint movements using marker-based motion capture system, muscle activations using surface electromyography, and metabolic cost using a portable indirect calorimetry device (see Fig. 1).

In summary, the unique features of the presented dataset, publicly available on figshare^49^ include:

- **comprehensive measurement data** – our dataset contains four different types of data that have commonly been used in characterising human walking: joint kinematics, ground reaction forces, muscle activity, and metabolic cost data, a combination rarely available in other studies;
- **hundreds of strides per condition** – unlike many datasets that only contain several strides per condition, our dataset contains 5-min worth of strides per condition, allowing analysis of gait parameter acclimatisation (and plateauing) over time as well^50^;
- **internal constraints** – in addition to studying the effects on gait of changing external constraints (cadence, walking speed), our dataset also imposes internal constraints (limited joint angle), providing insights into *how* (through recorded adaptations) and *why* (through metabolic, energetic, and biomechanical variables) humans walk the way they do in tested conditions/environments; and
- **individual level adaptations** – unlike studies that speed/age/sex-match their control and intervention groups and are thus limited to trends and group averages, our dataset allows studying individual adaptations across many different conditions.

## Methods

### Participants

Neurotypical adults were invited to participate in the study through a public advertisement made available on the official website of the Human Robotics Research Group of The University of Melbourne, Australia. The advertisement described the study goal, protocol, and duration and provided communication channels for those interested in knowing more and/or joining the study. The interested participants were interviewed and formally recruited if they met a set of inclusion/exclusion criteria. Inclusion criteria were (i) neurotypical young persons with no known lower limb impairments, (ii) 18-60 years old, (iii) 1.55-1.90 m tall, and (iv) weighing between 50-90 kg. Exclusion criteria were (i) significant trauma to the lower limbs and/or orthopedic procedures in the last six months, (ii) any form of diagnosed gait abnormalities and/or walking problems, (iii) cognitive and/or communicative problems affecting the ability to comprehend or follow instructions, and (iv) any other pain conditions.

Following this, 21 neurotypical young adults (5 females, 30 ± 8 years, body mass 72.7 ± 12.3 kg, height 1.72 ± 0.09 m, BMI 24.5 ± 2.7 kg/m^2^, mean ± standard deviation) participated in the study. Of those, 19 completed the entire protocol, and two dropped out after two out of three sessions (their data are also available with the manuscript). The anthropometric details of all the participants are summarised in Table 1. Only one participant (Sub3) had previous experience walking with a lower limb orthosis, albeit in a different study. All participants were provided a detailed plain language statement and signed the informed consent form that included a clause on agreeing to use pictures/videos in data dissemination. The ethics committee of The University of Melbourne approved the study (2021-20623-13486-3).

**Table 1:** Anthropometric data of the study participants.

Sub ID	Age [years]	Height [m]	Weight [kg]	BMI [kg/m^2^]	Sex (M/F)	Leg len. [m]	Cadence [step/min]	Pref. walk speed [m/s]	Treadmill experience	Physical activity
S1	35	1.72	74	25.0	M	0.87	104	1.12	Single-belt	Walking
S2	33	1.74	70	23.1	M	0.89	94	1.00	Dual-belt	Gym
S3	33	1.86	86	24.9	M	0.97	105	1.40	Dual-belt	Various
S4	25	1.75	76	24.8	M	0.87	109	1.00	Dual-belt	Various
S5	32	1.68	51	18.1	M	0.82	112	1.05	Single-belt	Tennis
S6	43	1.73	80	26.7	F	0.91	107	1.21	Single-belt	Walking
S7	27	1.67	64	22.9	M	0.86	109	0.94	No	None
S8	24	1.76	84	27.1	M	0.91	105	1.20	Dual-belt	Gym
S9	23	1.60	65	25.4	M	0.82	99	1.15	Single-belt	None
S10	22	1.88	77	21.8	M	0.95	95	1.05	Single-belt	Walking
S11	24	1.75	90	29.4	M	0.95	103	1.20	Single-belt	Gym
S12	26	1.74	82	26.9	M	0.84	105	1.10	Single-belt	None
S13	29	1.73	82	27.4	M	0.87	98	0.96	Single-belt	Running
S14	30	1.72	79	26.7	M	0.87	91	0.94	Single-belt	Walking
S15	27	1.55	50	20.8	F	0.78	114	0.98	Single-belt	Gym
S16	28	1.55	51	21.2	F	0.84	121	1.23	Single-belt	Gym
S17	29	1.65	72	26.4	F	0.84	105	1.10	Single-belt	Gym
S18	25	1.80	80	24.7	M	0.93	105	1.25	Single-belt	Gym/Run
S19	57	1.56	59	24.2	F	0.74	104	1.03	Single-belt	Walking
S20	29	1.74	66	21.8	M	0.93	96	1.10	Single-belt	Jogging
S21	26	1.85	88	25.7	M	0.96	95	1.01	Single-belt	Martial arts

Leg length was measured from the frontal pelvic marker (ASIS) to the lateral ankle marker (LM) – see *Data collection* section for more details. BMI is a body mass index calculated as the ratio of weight in [kg] and squared height in [m^2^]. Cadence refers to the preferred step frequency of walking at a preferred speed (measured during the preliminary session (*Ses1*) using a staircase method and indicated in the subsequent column). Treadmill experience refers to any previous use of a treadmill, including Single Belt (found in a gym) and Dual Belt (found in research labs). Physical activity serves as a proxy for the participants’ physical conditioning. In terms of Sex, M = male and F = female.

### Experimental conditions

The experimental study was designed around three multidimensional factors: walking speed, step frequency (i.e., cadence), and physical constraint. Walking speed came in three levels, including slow (*v*_1_ = 0.4 m/s), medium (*v*_2_ = 0.8 m/s), and normal (*v*_3_ = 1.1 m/s). Step frequency came in five different levels, including preferred (*f*_3_), two lower (*f*_1_ = 0.9*f*_3_ and *f*_2_ = 0.95*f*_3_), and two higher (*f*_4_ = 1.1*f*_3_ and *f*_5_ = 1.2*f*_3_) levels. Finally, physical constraint came in two levels, including a condition without restrictions on the lower limbs (*c*_1_) and one with a unilateral knee orthosis (*c*_2_, shown in Fig. 1). The orthosis was always worn on the left side with the intention of locking the participants’ knee joint in full extension. Each participant underwent all 30 combinations of the three factors (3 × 5 × 2) spread over two data collection sessions (see next section for details). Table 2 gives an overview of the naming convention used in the data analysis (and found in the dataset shared on figshare). The choice to go with asymmetric step frequency above and below the preferred one was made after preliminary testing showed that 0.8*f*_3_ (i.e., − 20%) at slow speed leads to highly imbalanced and potentially dangerous walking. As mentioned, all testing was done on a dual-belt instrumented treadmill in the Motek CAREN lab installed at the biomechanics lab at The University of Melbourne.

**Table 2:** The naming convention used in data analysis.

Speed / cadence	Walking without orthosis (c1)					Walking with orthosis (c2)				
	− 10%	− 5%	pref.	10%	20%	− 10%	− 5%	pref.	10%	20%
1.1 m/s	T1	T2	T3	T4	T5	T16	T17	T18	T19	T20
0.8 m/s	T6	T7	T8	T9	T10	T21	T22	T23	T24	T25
0.4 m/s	T11	T12	T13	T14	T15	T26	T27	T28	T29	T30

Tests *T1-T15* refer to free walking (i.e., *c*_1_), grouped in bouts of five for a given walking speed, while tests *T16-T30* refer to their counterparts in the case of constrained walking (i.e., *c*_2_). The order of tests was randomised for each participant, as described in section ‘Data collection session (Ses2/Ses3).’

### Experimental protocol

The study was organised into *sessions*, *bouts*, and *conditions* in the specified order of complexity. The participants were asked to come into the lab on three different days, each corresponding to one session. The first session (*Ses1*) served as a preparation and familiarisation day, while the second (*Ses2*) and third (*Ses3*) sessions were data collection days.

**Fig. 2:**
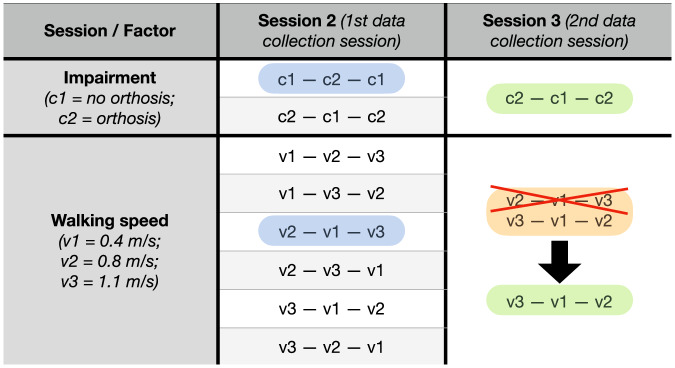
Example experimental conditions for one participant. To avoid familiarisation effects, only two orders of impairment and six of speed were available to choose from (middle column, full list). Then, a unique speed-impairment combination was chosen randomly during the first data collection session (*Session2*), highlighted in blue. This left one impairment option for the second data collection session (*Session3*), highlighted in green. The *inverted* impairment order across the two sessions left only two walking speed orders to choose from (highlighted in orange) to ensure all speed-impairment combinations across sessions. However, to avoid the order-of-speed bias that would occur if the speed order remained the same across sessions, only one speed combination remains available, highlighted in green.

The two data collection sessions were organised into 3 bouts each, with bouts differing in a unique *v*_*i*_ − *c*_*j*_ speed-constraint factor combination. A bout consisted of 5 walking conditions executed at the same walking speed with no breaks in between. With that, each participant underwent 15 (half) walking conditions per session, reducing the potential impact of fatigue while still collecting large amounts of data. The order of the cadence factor levels (i.e., *f*_1_ to *f*_5_) within a single *v*_*i*_ − *c*_*j*_ speed-constraint factor combination (bout) was always randomised. On the other hand, the order and distribution across sessions of bouts were only *partially* randomised to avoid familiarisation effects. For example, multiple occurrences of the speed (e.g., *v*_1_ − *v*_3_ − *v*_1_) or consecutive occurrences of the impairment factor (e.g., *c*_1_ − *c*_2_ − *c*_2_) within a single session were rejected, reducing the number of randomisation outcomes. This was further extended by the requirement to avoid the order-of-speed bias, leading to a uniquely determined choice of the speed-constraint factor combinations available to choose from. Fig. 2 gives an example of the conditions randomisation for a single participant.

#### Preparatory session (Ses1)

**Fig. 3:**
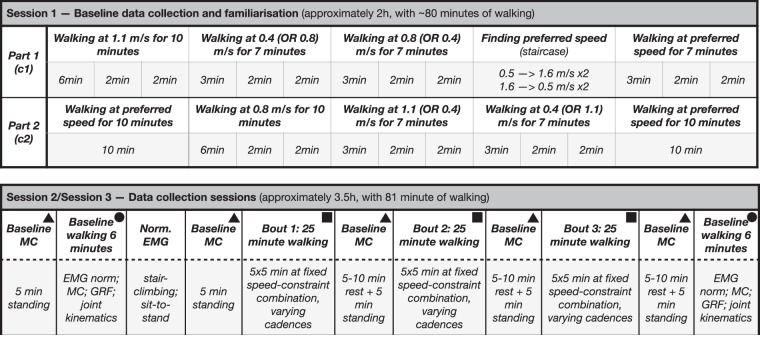
Experimental study protocol. (*Top*) After their baseline measurements were taken, participants walked at all three walking speeds (Part 1) to familiarise themselves and to find their speed-specific preferred cadence. The preferred cadence is an average of three numbers: one measured in the last 15 seconds of steady walking (6 min or 3 min) and two in the last 15 seconds of the 2-minute exploration periods. After finding their preferred speed using the staircase method, participants repeated the same 7-minute procedure (3+2+2) at their preferred speed. No rest was given between these tests (unless the participant specifically asked for it). The entire block was repeated with the orthosis and slight modifications (Part 2), resulting in a total of approximately 80 minutes of walking. (*Bottom*) The data collection sessions started with a baseline MC measurement, followed by a 6-minute walking test at the preferred speed. The same 6-minute test was repeated at the very end of each session, serving as a warm-up (start) and a baseline (start and end). Walking tests were always separated by a short 5-10 minute break (longer only if requested by the participant), and a resting MC was measured before each walking test. Participants were warned using auditory cues about the upcoming change in cadence to ensure they were aware of the change and alerted by the investigators if they started missing metronome beats. Across the two sessions, participants walked at each step frequency at all three walking speeds twice - with and without the orthosis. Each participant ended up walking twice with the orthosis in one session and once in the other, in a randomised order. Note: the same activities are symbol-labelled for easier understanding.

The first session served as a study familiarisation and baseline day. The participants started by walking at 1.1 m/s and preferred cadence for six minutes^50^ (Fig. 3, top - Part 1). The preferred cadence was estimated during the last 15 seconds of that period using a simple script that counts heel strikes in real-time. This number was then used to guide two subsequent 2-minute exploration periods at the same speed. During the first 30 seconds of each exploration period, a metronome guided participants 20% higher or lower from their previously estimated preferred cadence in a randomised order. The participants were then allowed to go back to their preferred cadence for the remaining 90 seconds in each 2-minute period. The same was repeated at 0.4 and 0.8 m/s walking speeds in a randomised order, albeit with a shorter, 3-minute familiarisation period (as opposed to a 6-minute period at 1.1 m/s). This allowed the calculation of preferred cadences based on three experimentally measured values^51^. The preferred cadence at each speed, calculated as an average of the three measured values, is given in Table 3.

**Table 3:** Preferred cadence at each of the three walking speeds.

	0.4 m/s	0.4 m/s	0.8 m/s	0.8 m/s	1.1 m/s	1.1 m/s
	(c1)	(c2)	(c1)	(c2)	(c1)	(c2)
**S1**	58	62	86	86	104	100
**S2**	57	59	83	83	99	99
**S3**	52	59	76	78	95	90
**S4**	73	74	92	91	107	103
**S5**	81	86	101	101	114	114
**S6**	52	60	82	84	101	101
**S7**	93	/	105	/	111	/
**S8**	70	75	89	86	102	101
**S9**	56	59	81	80	99	94
**S10**	63	75	83	92	97	101
**S11**	70	72	91	83	101	91
**S12**	72	75	93	94	106	108
**S13**	68	60	90	85	101	95
**S14**	57	59	81	87	96	97
**S15**	71	70	107	97	117	116
**S16**	78	90	106	98	116	111
**S17**	69	61	95	84	105	98
**S18**	56	60	77	79	97	94
**S19**	68	66	107	118	111	117
**S20**	59	54	82	78	96	92
**S21**	52	59	84	77	99	93

The resulting value is an average of three experimentally obtained values. Participant S7 was unable to walk with the orthosis. *c1* = free and *c2* = constrained walking.

The participants were then instructed to find their preferred walking speed using the staircase method^52,53^. In short, the treadmill speed was twice increased from 0.5 m/s and decreased from 1.6 m/s in 0.1 m/s increments (every 10 seconds), until participants identified their preferred speed. The initially identified value was fine-tuned by changing the speed in 0.05 m/s increments up or down until the person settled into the final value for that segment. This speed was noted 4x and averaged to obtain the final preferred speed. This was followed by a 7-minute walking test at that preferred speed, similar to the tests at the other three speeds as described above, with the aim of finding the preferred cadence at the participant’s preferred speed.

The process of finding each person’s preferred step frequency (cadence) at each of the three speeds was subsequently repeated while wearing the orthosis (Fig. 3, top - Part 2). Similarly, the preferred cadence was estimated during the last 15 seconds of three subsequent periods using a simple script. The main difference between the free (Part 1) and constrained (Part 2) walking parts lies in how a preferred speed is found. Unlike the staircase method used during free walking, a simple increase in the treadmill speed until participants provided an auditory cue was used during constrained walking. This is because constrained preferred speed is not used in data collection sessions, thus making finding its precise value less critical. The main reason for still finding the preferred speed during constrained walking was to allow participants to familiarise themselves with the orthosis by giving them more time to walk with it. Once the self-selected speed was identified, which usually took a couple of minutes, all participants were given 20 minutes of walking with the orthosis at the identified speed, split into two 10-minute walking periods at the start and end of Part 2 (see Fig. 3).

#### Data collection session (Ses2/Ses3)

During the two data collection sessions, participants walked across three speed-constraint factor combinations (i.e., bouts), each taking 25 minutes (Fig. 3, bottom). Without a break in between different cadences (1 cadence = 1 test = 5 min), each bout had speed and impairment fixed while sweeping through all five step frequencies (*f*_1_ to *f*_5_) in a randomised order. A metronome guided participants to ensure the desired step frequency, a practice shown not to affect the metabolic cost (MC)^51^. The test length of 5 minutes is based on a previous study that demonstrated that it takes about 3 minutes for participants to reach 95% of their metabolic steady-state^3^, leaving two minutes’ worth of data for averaging (one in a more conservative case).

The three bouts were separated by a 5-10 minute break and another 5 minutes to measure resting metabolic cost while standing. In total, the resting MC was measured five times throughout each session to capture a potential increase in resting energetic demands as the session proceeds and thus validate the metabolic-level fatigue effects. The effects of potentially accumulating fatigue were also measured using EMG amplitude signal (in addition to MC) during walking at a preferred speed, a 6-minute test repeated at the start and end of each session. The former (i.e., muscle-level fatigue) can be validated using different methods in both time and frequency domains^54^.

In terms of the equipment, participants were fitted at the start of each data collection session with retroreflective markers that capture their lower limbs (and in some cases, upper body) movement in space, wireless surface electromyography (EMG) to measure their EMG amplitude (an estimate of muscle activity), and a portable indirect calorimetry system to measure their energy use. An experienced postdoctoral researcher, trained in placing the equipment and having previously led two human studies, put all sensors and markers on for all participants. The first 6-minute walk at the preferred speed served as a warm-up trial^50^ and to control the potential fatigue effects. The test was immediately followed by ten repeated motions of sitting down and standing up (about knee height chair), and two passes up and down a flight of 15 stairs. The muscle activity measured during these two tests and a 6-minute walking can be used in finding a muscle activity normalisation factor^55^ (see later sections for more details).

Participants were asked to comply with the following before coming into the lab: (i) no strenuous exercise at least 12 hours, preferably 24 hours before coming into the lab, (ii) no alcohol, caffeine, or nicotine in the period of 4 hours before the session start, and (iii) large meal no later than 1.5 hours before coming into the lab. Participants were only allowed to drink water during the session, and no food was permitted.

#### Walking speeds

The *normal* walking speed was initially set to 1.25 m/s as this speed is commonly used in tests with healthy participants^56–60^ and is considered the preferred average walking speed in young adults^9,13^. However, walking with the orthosis at that speed and +20% step frequency proved to be potentially dangerous for the participants, so a decision was made to reduce this to 1.1 m/s.

The other two speeds were chosen to facilitate healthy-patients walking comparison, acknowledging that differences between the two groups go beyond simply speed-matching their gaits. The 0.8 m/s is considered a lower boundary of the so-called community ambulator category^61^ and is typical of high-functioning hemiparetic patients^25,29,34,62^. The 0.4 m/s is considered an upper boundary of the so-called household ambulator category^61^ and is typical of low-functioning hemiparetic patients^25,63,64^.

### Unilateral knee orthosis

**Fig. 4:**
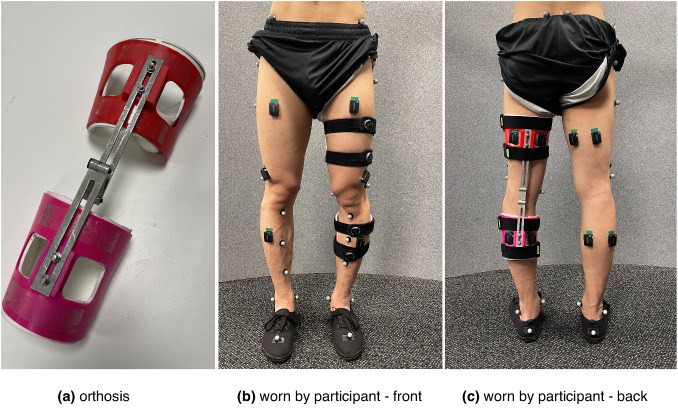
Knee orthosis used in the study. **(a)** Multiple cuff sizes, adjustable cuff position with respect to the knee joint, and double-hinge design allowed adjustments and a comfortable fit for multiple participants. A 4 mm thick Softair White (Massons Healthcare, VIC, Australia) padding is placed on the cuffs' inner side for further comfort. The orthosis weighs about 250g. **(b,c)** Orthosis worn by a subject, front (**b**) and rear (**c**) view. The orthosis was fixed in place by four BOA straps, which made donning and doffing of the orthosis simple and quick while not compromising on a tight fit for the participants. Cutouts in the plastic cuff allow the placement of EMG sensors and retroreflective markers. All equipment was placed by the same person, the lead investigator, who has extensive experience in running human studies.

A simple, in-house designed orthosis/brace was used to lock the knee joint and elicit compensatory movements during walking (Fig. 4a). The orthosis consists of two 3D-printed cuffs and two metal bars with a double-hinge joint connecting the cuffs. The cuffs are based on a 3D model of several human legs obtained using a 3D scanner and imported into 3D modelling software. The cuffs come in several sizes to account for variations in the participants’ legs. Furthermore, the design allows changing the cuffs’ relative position with respect to the knee joint to provide a better fit and comfort.

For consistency, participants always wore the orthosis on their left leg (Fig. 4b,c). To allow the placement of retroreflective markers for the motion capture system and EMG sensors, cuffs come with multiple cut-outs. The cuffs were secured in place by four BOA straps in total, two on each cuff. A double-hinge system allows adjusting the orthosis to different leg sizes and changing the fixed knee angle. In this study, the fixed knee angle was set by locking the hinge when a participant was standing with their knee fully extended, rendering a zero biological knee angle in a static pose. During experiments (i.e., dynamic movements), the compliant tissue in human legs inevitably allows a small amount of knee flexion in the swing phase of gait. As a consequence, the effective knee flexion during walking was always higher than zero (up to 20^°^ in the swing in some participants). The orthotic movement did not cause any discomfort or pain to participants, likely due to the soft padding lined on the cuff’s inner side.

### Data collection and processing

Four categories of data were collected in the study: lower limb kinematics using a marker-based motion capture system (in the case of half of the participants also upper limb, see Table 4), ground reaction forces (GRF) using a force-instrumented dual-belt treadmill, an estimate of muscle activation using wireless surface electromyography (EMG) sensors, and the metabolic cost of walking using indirect calorimetry. All data processing was done using custom-written scripts in Matlab 2021a (Mathworks, Massachusetts, USA).

**Table 4:** Marker data availability across participants.

	S1	S2	S3	S4	S5	S6	S7	S8	S9	S10	S11	S12	S13	S14	S15	S16	S17	S18	S19	S20	S21
**Lower limb**	26	24	24	24	24	24*	24	26	26	26	26*	24	26	26	26	26	26	26	26	26	26
**Upper limb**	**x**	9	11	**x**	**x**	11	**x**	11	11	11	**x**	**x**	11	11	11	**x**	11	11	**x**	**x**	**x**

All participants wore a set of markers on their pelvis and lower limbs. Upper limb (torso) markers were optional, with just over half the participants (11/21) agreeing to wear extra markers on their torso. If the participant wore no markers on the upper limbs, **x** is used. (*To note: Participant S6 had 24 lower limb markers in Session 2 and 26 in Session 3, differing in TIBAux2 markers added in Session 3; S11 had 24 markers during preferred walking in Session 2 and 26 in all other tests, with the same change of markers as in S6.)

#### Kinematics and forces data

**Fig. 5:**
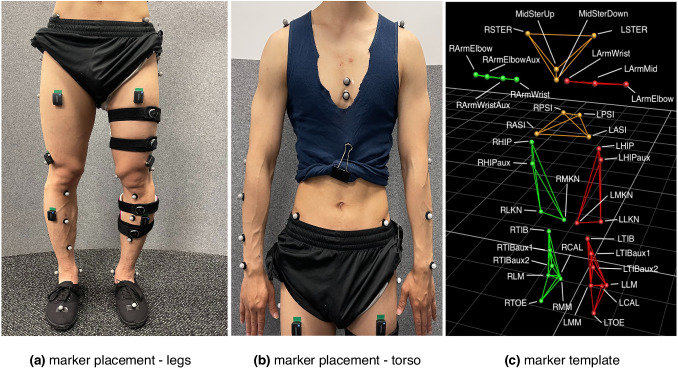
Marker placement. **(a,b)** Markers on one participant. Participants wore clothes that bare their legs and pelvis area, and, if torso markers are used, their shoulders, arms, and top of the chest. **(c)** Marker template defined in Vicon Nexus software. Marker clusters are colour coded, with the left and right sides coloured in red and green, respectively, and the pelvis and sternum in orange. The setup consists of functional and auxiliary (denoted by the *aux* suffix) markers, the latter being used to differentiate the two sides of the body by introducing asymmetry into the marker template (e.g., four markers on the right arm vs. three markers on the left arm). All but two markers (MidSterUp and MidSterDown) start with either *L* or *R*, denoting the left and the right side, respectively. The meaning of acronyms (bar the first letter, L or R), is: STER = sternum, PSI = posterior superior iliac spine, ASI = anterior superior iliac spine, LKN = lateral knee, MKN = medial knee, TIB = tibia, LM = lateral malleolus, MM = medial malleolus, CAL = calcaneous.

Gait data was collected in the Motek CAREN (Motek Medical B.V., the Netherlands) lab environment using a dual-belt instrumented treadmill and Vicon motion capture system with 10 cameras (Vicon Motion Systems, Oxford, UK). The marker data was collected at 100 Hz, and force data (GRF) at 1 kHz. Participants were fitted with a custom set of 26 retroreflective markers (spherical, 14 mm diameter, B&L Engineering, CA, USA) bilaterally placed in the pelvic area and on lower limbs. Of the 26 markers used, 23 are functional following^65^, and the remaining three are added to allow differentiating the two legs through asymmetric marker patterns. Half the participants also agreed to have markers placed on their torso and arms, in which case a custom set of 11 markers was used (Fig. 5a,b). It should be noted that while 26 lower limb and 11 upper limb markers were the default marker configuration, some participants had 24 lower limb markers and 9 upper limb markers, with two auxiliary markers missing in both cases. This was the case with a few early participants and is indicated in Table 4. The two missing markers include *LTIBaux2* and *RTIBaux2* in the case of lower and *RArmWristAux* in the case of the upper limb.

Data were segmented into steps using a 5% GRF signal threshold (i.e., for a 70 kg person, the threshold is 70 ⋅ 9.81 ⋅ 0.05 = 34*N*), as this was the lowest threshold across all participants that had a negligible amount of false positives. False positives occur when the force oscillates around the defined threshold, resulting in, e.g., multiple heel strikes detected in a single step. The provided gait segmentation algorithm comes with a code for the automated correction of false positives. The code also corrects for the missing steps, which occur when a person steps on the opposite belt (i.e., left leg on the right belt, called overstepping). Segmented and corrected data were subsequently time-normalised to 0-100% using linear interpolation, with the step defined as heel strike to subsequent heel strike. The number of detected steps on each side (and consequently, the number of heel-strike and toe-off events) is not necessarily equal in the raw data and was thus corrected in post-processing for the simplicity of the subsequent analysis. Furthermore, the steps were removed if/where needed at the start/end of a dataset (i.e., a 5-minute test) to ensure every dataset starts with the left and ends with the right step (again, for the analysis’ simplicity).

The marker data were filtered using a 4th-order zero-lag Butterworth filter with a 6 Hz cut-off frequency^9^. Filtered marker coordinates in 3D space of lower limbs were used to calculate joint angles (ankle, knee, hip, and pelvis) in the sagittal plane as per Research Methods in Biomechanics^66^ and following ISB (International Society of Biomechanics) guidelines^67^. Torso markers are only available for half the participants but do not allow for a similar calculation of the upper limb joint angles – their purpose was to capture arm swing movements and chest movements rather than upper limb joint angles. The chest movement is particularly useful in calculating, e.g., Lyapunov stability index^52,68^, an increasingly relevant index in the patient population. A standing static calibration trial was performed at the start of each session to build a marker template and define *offset* joint angles. These angles capture imperfections in placing markers (e.g., a measured 1^°^ knee flexion despite the person standing with their knees fully extended) and were subtracted from dynamic movement to obtain a true measurement of the joint angle (i.e., walking ROM) rather than errors in marker placement. During the static trial, participants were instructed to stand still, hip-width apart with straight knees and back and feet parallel with toes facing forward, as seen in Fig. 5c^66^. Study investigators demonstrated this position to each participant and ensured they followed the instructions. Once calculated for the entire test, kinematic data were segmented into gait cycles using heel strike and toe-off event indices determined using GRF signal.

#### EMG amplitude data

The EMG amplitude was measured in eight muscles per leg using surface electromyography (sEMG) Delsys Trigno (Delsys Inc., Natick, MA, USA) system. The raw data signal, captured at 2 kHz, was read by the Vicon PC running Nexus 2.8 via cable connection for data synchronisation purposes. For participants S1-12, the connection was done using a USB cable (trigger not available), which Delsys claims results in a synchronisation error of ± 20 ms on average (in our experience, the variations can significantly exceed this estimate). For participants S13-21, a trigger was available to ensure no-delay synchronisation. NOTE: The delay was corrected in participants S1-12 in post-processing by cross-correlating accelerometer data from the Right Tibialis Anterior EMG/IMU sensor with accelerations from RTibExt marker data, a method validated on the synchronised data from participants S13-21. The correction (in ms) for each participant is given in Supplementary File, Table S1. A validation of this approach for correcting delay is demonstrated in Supplementary File, Fig. S1.

The wireless Trigno Avanti sensors were placed in line with muscle fibers^69^ over the Tibialis Anterior (TibAnt), Gastrocnemius Lateralis (GastroLat) and Medialis (GastroMed), Vastus Lateralis (VastLat) and Rectus Femoris (RecFem), Biceps Femoris (BicFem) and Semitendinosus (Semitend), and Gluteus Maximus (GlutMax) muscles (Fig. 4a,b). The same order of muscles is preserved in raw files provided with the dataset, with muscles 1-8 (EMG sensors 1-8) corresponding to the left leg and 9-16 (EMG sensors 9-16) to the right leg. NOTE: Data are organised per muscle name and not per sensor name.) Each participant’s skin was prepared following the SENIAM guidelines (website, 2023). This included shaving the hair in the spots of the sensor placement, as well as cleaning the skin with alcohol and ensuring it was dry before the sensors were attached.

The processing of the raw EMG data started by detecting a linear envelope. This was done using a band-pass filter (10-500 Hz, implemented on the Trigno sensors) followed by a full-wave rectification and smoothening (a 100 ms moving average window). Linear-envelope data were normalised using pooled EMG activity collected during stair climbing, sit-to-stand activity, and baseline walking test at the start of each session. Maximal muscle activity was determined in each muscle per person per activity, and the highest value across the three tests per muscle was used as a normalisation factor (see Table S2 in Supplementary File). The reason for not using Maximal Voluntary Isometric Contraction (MVIC) tests was the lack of necessary equipment to correctly measure maximum EMG amplitude and to avoid major drawbacks of the isometric contraction approaches^55,70^, including inconsistent maximal contractions and inability to volitionally contract specific muscles. The activity-based method used herein also has the benefit of ensuring normalised muscle activity to be between 0 and 1 (muscles are generally more active in stair climbing and sit-to-stand movement than walking), which is a prerequisite for using these data in driving musculoskeletal models (e.g., OpenSim). Other ramifications of this approach can be found in the section Usage Notes. Muscle activity data were segmented into gait cycles using heel strike and toe-off event indices determined using GRF signal. Additionally, a signal-to-noise (SNR) ratio, calculated as 20 ⋅ *log*10(*signal*_*rms*_/*noise*_*rms*_), is provided for each muscle across all gait cycles (similar to segmented EMG amplitude). Observed noise levels were predominantly in the ( − 5, 5)*μV* and ( − 3, 3)*μV*_*rms*_ range, so the latter is used in the provided SNR calculations. Whenever SNR > 6 dB, which corresponds to the RMS amplitude of the actual EMG signal that is more than twice the RMS amplitude of the noise, the signal is considered good. NOTE: Normalisation factors of each activity (walking, stair climbing, and sit-to-stand) can be extracted and used separately from the provided raw EMG data of walking, stair climbing, and sit-to-stand activity.

#### Metabolic data

Respiratory *O*_2_ consumption and *CO*_2_ production were collected using PNOE indirect calorimetry system (Endo Medical, Palo Alto, US), consisting of an appropriate face mask and a portable measurement unit worn on the back. The masks come in different sizes to accommodate different participants and minimise the exchanged gas leakage. The data of the last minute of both 5-minute resting (standing still) and 5-minute walking tests were used to calculate the energy consumption using the simplified Brockway formula^71^. Net MC of walking (in *W*/*kg*) was then calculated by subtracting the resting value measured before each bout from all 5-minute tests within that bout and dividing by the participant’s mass. This was done to avoid the effect of potentially accumulating fatigue reflecting in the net energetic cost of the tests later in the session. Furthermore, the metabolic cost of transport (in *J*/*kgm*) was calculated by dividing the normalised MC by the walking speed. NOTE: Resting MC throughout each session is provided with the dataset.

## Data Records

**Fig. 6:**
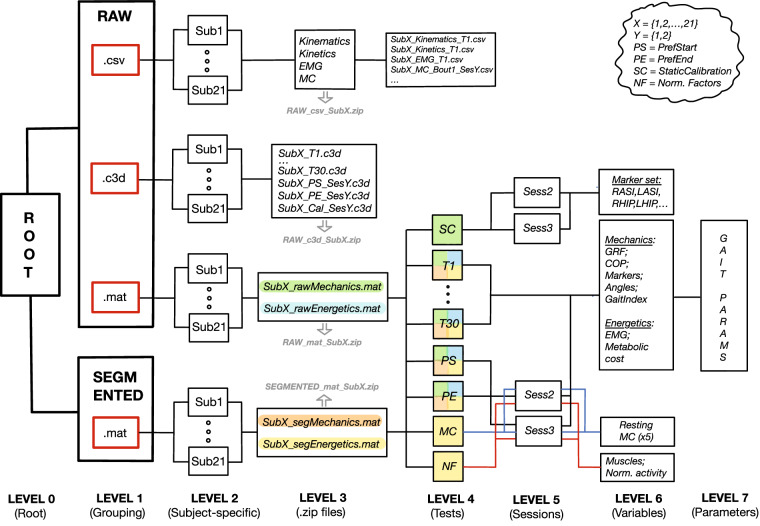
Schematic representation of the provided dataset. The data are organised into multiple levels. The first three levels 0-2 contain the main structure, level 3 is where the actual .zip files uploaded to figshare reside, and levels 4-7 further divide dataset into tests and all relevant gait variables. Overall, the dataset is grouped into segmented and raw data on Level 1 and organised per subject on Level 2 for easy navigation. Four .zip files per person are available on Level 3, including three raw data files (*RAW_csv_SubX*, *RAW_c3d_SubX*, and *RAW_mat_SubX*) and one segmented data file (*SEGMENTED_mat_SubX*), where *X={1,2,...,21}*. Level 4 contains .csv files of all the tests across Sessions 2 and 3 in *RAW_csv_SubX.zip* file, and between 30 and 32 fields in the structure in *RAW_mat_SubX.zip* file (*RAW_c3d_SubX.zip* does not go beyond Level 3). MAT fields in the structure on Level 4 are color-coded with respect to Level 3 for easier navigation (e.g., every field on Level 4 with a green mark in the box is contained in *SubX_rawMechanics.mat* file from Level 3). Five fields from Level 4 in segmented and raw .mat files have session-specific sub-fields *Sess2* and *Sess3* on Level 5. These include Static Calibration (SC), Preferred Walking at the Start (PS) and End (PE) of a session, Metabolic Cost (MC), and EMG Normalisation Factors (NF). The latter two have colour-coded connections across Levels 4 and 6 for correct field assignments. All walking test fields on Level 4 (T1…T30, PS, PE) contain relevant variables on Level 6 (e.g., GRF, CoP, joint angles, etc.). MC contains data from the five standing trials per session when resting MC was measured, and NF an activity used in normalisation (e.g., walking) per muscle and the max signal value measured in that activity. For simplicity, Gait Parameters from Level 7 are given in Table 5.

The full dataset is available on figshare^49^. Since figshare repository does not allow folder structure at the time of submission, all data are uploaded as .zip files, grouped according to the content organisation depicted in (Fig. 6 and Table 5). More details are provided in the subsequent sections. Where possible, the same naming and data structure conventions are maintained across different data groups for consistency.

**Table 5:** Gait parameters from Level 7 in the provided dataset (see Fig. 6).

Gait Variables (Level 6)	Gait Parameters (Level 7)	Description
GRF (Ground Reaction Forces)	V_Left; V_Right; AP_Left; AP_Right; LM_Left; LM_Right	GRF in all 3 planes, separated per leg; raw in [N], segmented in [N/kg]
CoP (Centre of Pressure)	AP_Left; AP_Right; LM_Left; LR_Right	Location of the resultant GRF in x-y plane [mm]
Markers	Markers available for that subject (e.g., RASI, LTO, LLKN, RTIB)	Location of each marker in x-y-z (global) planes [mm]
GaitIndex	LHS; RHS; LTO; RTO; LHS_1kHz; RHS_1kHz; RTO_1kHz; LTO_1kHz	Gait indices in 100 Hz and 1 kHz resolution
Angles	Hip_Left; Hip_Right; Knee_Left; Knee_Right; Ankle_Left; Ankle_Right; Pelvis_Sagittal; Pelvis_Frontal; Pelvis_Transverse	Pelvis angles in all three planes; hip, knee, and ankle angles in the sagittal plane [deg]
EMG	Left_TibAnt; Left_GastroMed; Left_GastroLat; Left_VasLat; Left_RecFem; Left_BicFem; Left_Semitend; Left_GlutMax; Right_TibAnt; Right_GastroLat; Right_GastroMed; Right_VasLat; Right_RecFem; Right_BicFem; Right_Semitend; Right_GlutMax	EMG activity of all 16 muscles, separated per leg; raw in [mV], segmented as filtered/rectified/RMS in [mV]; signal-to-noise (SNR) ratio for all muscles, separated per leg and segmented into cycles; calculated as 20*log_10_(signal_RMS_/noise_RMS_)
MC	MetCost; CostTrans; RER	Metabolic cost of walking; MetCost in [W/kg] and CostTrans in [J/kg/m]; Respiratory Exchange Ratio (RER) [-]

The two sides are always separated by *Left* and *Right* in the parameter name, and the movement in three planes by *V* for vertical (z-axis), *AP* for anterior-posterior (y-axis), and *LM* for lateral-medial (x-axis). Full names of markers and muscles can be found elsewhere in this document. Gait indices are given in the marker data resolution (100 Hz) and GRF data resolution (1 kHz), and are labeled as follows: *LHS* and *RHS* for Left and Right heel strike, and *LTO* and *RTO* for Left and Right toe-off, all respectively. Raw EMG data are given in 2 kHz and segmented (filtered, rectified, and averaged) in 1 kHz.

### Raw data

CONTAINS: ground reaction force (GRF) and the feet centre of pressure (CoP) data; the 3D position of all the markers; muscle activity (EMG) data for each of the sixteen muscles; and metabolic cost (MC) data as exported by the metabolic analyser.

DESCRIPTION: Raw data are stored in .csv, .c3d, and .mat formats, all widely used and accepted file formats. The CSV format is a text format that can be read by any text file reader, including Microsoft Excel and Matlab. This is arguably the format that will reach the largest user base. The C3D format is typically used in the biomechanics community, but since it’s a binary format, it requires extra steps and processing before it can be accessed. Finally, raw data are also provided in MAT format that can be easily read in Matlab and Python, the two most commonly used programming languages in the research community. Where necessary, raw data files in CSV and MAT formats were *pre-processed* to, e.g., remove data from a 5-min test if the participant had to stop to fix a marker or reduce GRF frequency from 2 kHz to 1 kHz (an artifact of the originally collected GRF data frequency).

The *RawData* on Level 1 are organised into three groups: *CSV*, *C3D* and *MAT*, corresponding to the format the data are saved in. Within each group, data are separated per participant on Level 2, resulting in 21 sub-groups in each *CSV*, *C3D*, and *MAT* group. The data files available for download from figshare reside on Level 3 as .zip files. When downloaded and opened, the fields from Levels 4-7 will become available for further analysis and manipulation. In particular:

- **.csv** – files available for download are called, e.g., *RAW_csv_Sub17.zip*. Within this file, data are separated into four folders, corresponding to *Kinematics*, *Kinetics*, *EMG*, and *MC*. The *Kinematics* folder contains one CSV static calibration file per session, CSV files of all 30 tests (T1-T30), and two CSV files per session of preferred walking (at the start and end). Similar files can be found in the *Kinetics* and *EMG* folders, with the addition of CSV files for stair negotiation and sit-to-stand in the latter. The *MC* folder, on the other hand, contains three CSV files per session of 25-minute bouts, five CSV files per session of resting, and two CSV files per session of preferred walking. The file naming convention concatenates participant identification, type of data, test number, and session number where applicable. For example, marker data of S6 in T16 is located in *Kinematics* folder under name *Sub6_Kinematics_T16.csv* and will become available once the *RAW_csv_Sub6.zip* is opened. Similarly, MC data of S6 in Bout2 of Session2 is located in *MC* folder under name *Sub6_MC_Bout2_Session2.csv*.
- **.c3d** – files available for download are called, e.g., *RAW_c3d_Sub5.zip*. Since data are saved into a single C3D file per test, the names within the zip file are concatenated subject identification (e.g., Sub5) and test name (e.g., T17). For example, the raw data of S5 and test T17 is called *Sub5_T17.c3d* and will become available once the *RAW_c3d_Sub5.zip* file is opened. Apart from the C3D files of all 30 tests (T1-T30), each subject-specific folder also contains C3D files of static calibration (one per session) and preferred walking trials (two per session, one at the start and one at the end).
- **.mat** – files available for download are called, e.g., *RAW_mat_Sub13.zip*. To avoid too large file sizes, GRF, CoP, and markers data are grouped into *Mechanics*, and EMG and breath data into *Energetics*. The subsequent level in the tree structure is test names, followed by one of the data groups. For example, raw GRF data of S4 in test T16 is saved in *Sub4_rawMechanics.mat*, which will become available once *RAW_mat_Sub4.zip* is opened. Similarly, raw markers data of S20 in test T5 is saved in *Sub20_rawMechanics.mat* under the *rawMechanics.T5.Markers* field, which will become available once *RAW_mat_Sub20.zip* is opened. Marker positions during a static calibration trial for the same subject can be found under *rawMechanics.StaticCalibration* field in the same MAT file (separated into Session 2 and Session 3 data). Similarly, raw EMG data of S9 in T22 is saved in *Sub9_rawEnergetics.mat* under the *rawEnergetics.T22.EMG* field, which will become available once *RAW_mat_Sub9.zip* is opened. In addition to static calibration and data from 30 walking tests, each MAT file also contains raw data from the two walking tests at the preferred speed, divided into two sessions.

### Segmented data

CONTAINS: ground reaction force (GRF) and the feet centre of pressure (CoP) data; calculated joint and pelvic angles in all three degrees of freedom (DoFs); muscle activity data; calculated metabolic cost data (from breaths); heel-strike and toe-off events used in gait segmentation; and EMG normalisation factors including activities (e.g., walking) they were obtained from.

DESCRIPTION: Segmented data are saved in .mat format and can be accessed by opening *SEGMENTED_mat_SubX.zip* files. Similar to raw MAT files in *RAW_mat_SubX.zip*, all data is grouped per subject, resulting in 21 subject-specific groups. GRF, CoP, joint and pelvic angles, and heel-strike and toe-off events are grouped into *Mechanics*, while EMG data, EMG normalisation factors, and calculated metabolic cost are saved under *Energetics*. For example, segmented joint angles data of participant S19 in test T30 is saved in *Sub19_segMechanics.mat* under the *segMechanics.T30.Angles* field, while segmented EMG data of participant S8 in T11 is saved in *Sub8_segEnergetics.mat* under *segEnergetics.T11.EMG* field. Both .mat files will become available once relevant *SEGMENTED_mat_SubX.zip* files are opened. EMG normalisation factors for the same participant (i.e., S8) can be found under *segEnergetics.NormFactors* field in the same MAT file (separated into Session 2 and Session 3 data).

As mentioned earlier, data is segmented from the left heel-strike to the subsequent left heel-strike event, and time normalised to 101 data points. Data for each variable (e.g., hip joint angle, vertical GRF) are saved in matrices such that gait cycles always correspond to rows, and gait percentage to columns (1% of gait = 1 data point = 1 column). Except for pelvic angles and metabolic cost data, which are side-independent, all data is separated into the left and right sides. Furthermore, metabolic cost data is calculated on a breath-by-breath basis but is added to the segmented data folder as that seems appropriate.

### Missing data

The PNOE device used to measure *O*_2_ consumption and *CO*_2_ production malfunctioned in several sessions, resulting in partial data loss in the case of five subjects. These include participants S5, S6, S8, S11, and S12. An overview of the metabolic cost data across all participants and conditions (5-min tests) is given in Table 6.

**Table 6:** An overview of the MC data availability.

	1.1 m/s NO orthosis					0.8 m/s NO orthosis					0.4 m/s NO orthosis					1.1 m/s WITH orthosis					0.8 m/s WITH orthosis					0.4 m/s WITH orthosis				
	T1	T2	T3	T4	T5	T6	T7	T8	T9	T10	T11	T12	T13	T14	T15	T16	T17	T18	T19	T20	T21	T22	T23	T24	T25	T26	T27	T28	T29	T30
S*	ok	ok	ok	ok	ok	ok	ok	ok	ok	ok	ok	ok	ok	ok	ok	ok	ok	ok	ok	ok	ok	ok	ok	ok	ok	ok	ok	ok	ok	ok
**S5**	x	ok	x	x	x	ok	ok	ok	ok	ok	ok	ok	ok	ok	ok	ok	ok	ok	x	ok	ok	ok	ok	ok	ok	x	x	x	x	x
**S6**	x	x	x	ok	x	ok	ok	ok	ok	ok	ok	ok	ok	ok	ok	ok	ok	ok	ok	ok	ok	ok	x	ok	ok	ok	ok	ok	ok	ok
**S7**	ok	ok	ok	ok	ok	ok	ok	ok	ok	ok	ok	ok	ok	ok	ok	NA	NA	NA	NA	NA	NA	NA	NA	NA	NA	NA	NA	NA	NA	NA
**S8**	ok	ok	ok	ok	ok	x	x	x	x	x	ok	ok	ok	ok	ok	x	ok	x	ok	ok	ok	ok	ok	ok	ok	ok	ok	ok	ok	ok
**S11**	x	x	x	x	x	ok	ok	ok	ok	ok	ok	x	ok	ok	ok	x	x	ok	ok	ok	ok	ok	ok	ok	ok	ok	ok	ok	ok	x
**S12**	x	x	x	x	x	NA	NA	NA	NA	NA	ok	ok	ok	ok	ok	NA	NA	NA	NA	NA	ok	ok	ok	ok	ok	NA	NA	NA	NA	NA

The available data are indicated by an ‘ok’, and the missing by *x*. To save space, participants with all data available (i.e., S*) are grouped together, whereby S* includes S1-S4, S9, S10, and S13-S21. Two participants (S7 and S12) withdrew after the first data collection session (*Sess2*), and thus the missing data is indicated by *NA* (not applicable). Participant *S7* was unable to walk with the orthosis, which is why only the data in tests *T1-T15* are available.

A wrong setting was used with the Delsys proprietary data collection software (EMGWorks) when collecting muscle activity data during stair negotiation at the beginning of Sessions 2 and 3. For this reason, and only for participants S13-S21, EMG data is missing for the right Semitendinosus muscle in stair climbing. Consequently, this muscle’s activity was set to zero when using it in an activity-based normalisation process (i.e., column 15 in a 16-column EMG matrix is zero).

Further to issues with EMG data, the sensor measuring left Tibialis Anterior malfunctioned in Session 3 for participant S15. To keep the symmetry, some sensors were reshuffled, including moving the sensor from the left Gluteus Maximus to the left Tibialis Anterior and not measuring the former, as well as removing the sensors from the right Gluteus Maximus. In raw data files, this simply means that both left and right Gluteus Maximus are zero in all tests for participant S15 in Session 3. Note, however, that data for Left Tibialis Anterior is saved in raw files in column 8 in a 16-column EMG file, corresponding to Left Gluteus Maximus.

## Technical Validation

**Fig. 7:**
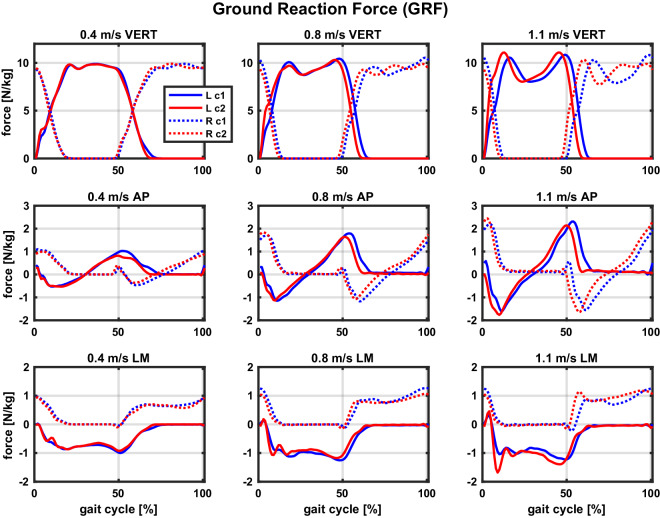
Example ground reaction forces in all three directions across conditions (taken from participant S2). Three walking speeds are separated into columns, and three planes of movement into rows (VERT = vertical; AP = anterior-posterior; LM = lateral-medial). Note the same y-axis limits per row to allow direct comparisons of force amplitudes across speeds. The free and constrained conditions are color-coded, with blue corresponding to the free (c1) and red to the constrained (c2) condition. Full lines correspond to the left leg (L) and dotted lines to the right leg (R). Data are taken from the tests with the preferred cadence at each speed. NOTE: Reader is referred to online version of this manuscript for colour-coding.

**Fig. 8:**
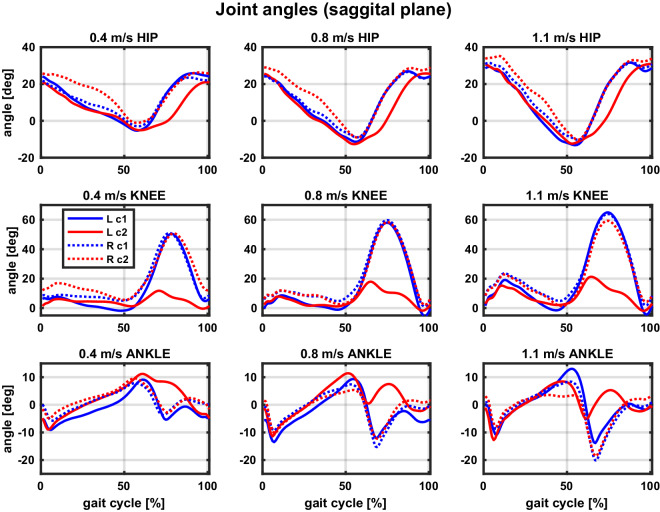
Example joint angles across conditions (taken from participant S8). Three walking speeds are separated into columns and three joints into rows. Note the same y-axis limits per row to allow direct comparisons of joint amplitudes across speeds. The free and constrained conditions are color-coded, with blue corresponding to the free (c1) and red to the constrained (c2) condition. Full lines correspond to the left leg (L) and dotted lines to the right leg (R). Positive angles correspond to flexion and negative to extension in all three joints. Data are taken from the tests with the preferred cadence. NOTE: Reader is referred to online version of this manuscript for colour-coding.

**Fig. 9:**
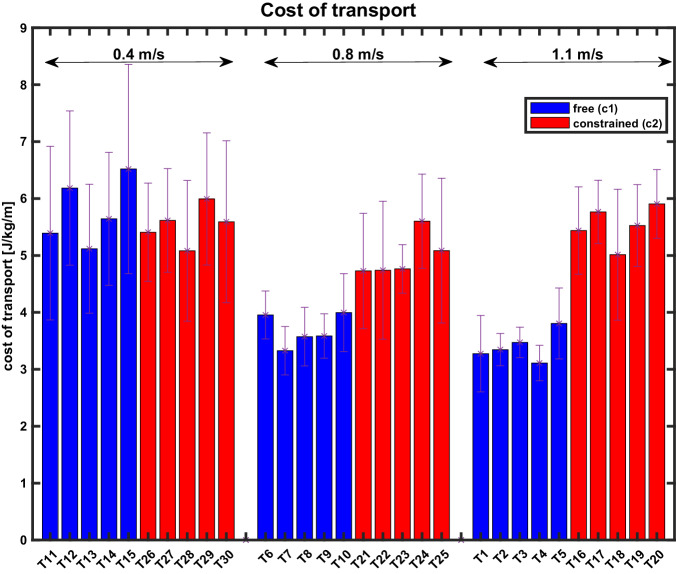
Example cost of transport across conditions (taken from participant S17). The data are grouped per speed, from left to right: 0.4 m/s to 0.8 m/s to 1.1 m/s, separated by empty vertical rows. Blue bars correspond to free (*c*_1_) and red to constrained (*c*_2_) walking conditions (similar to the color-coding of GRF and joint angles). The order of five bars per color per speed always corresponds to the same order of step frequencies, from left to right: − 10%, − 5%, preferred, +10%, +20% (see Table 2 for notation). Data are calculated as mean over the last minute of each test. NOTE: Reader is referred to online version of this manuscript for colour-coding.

**Fig. 10:**
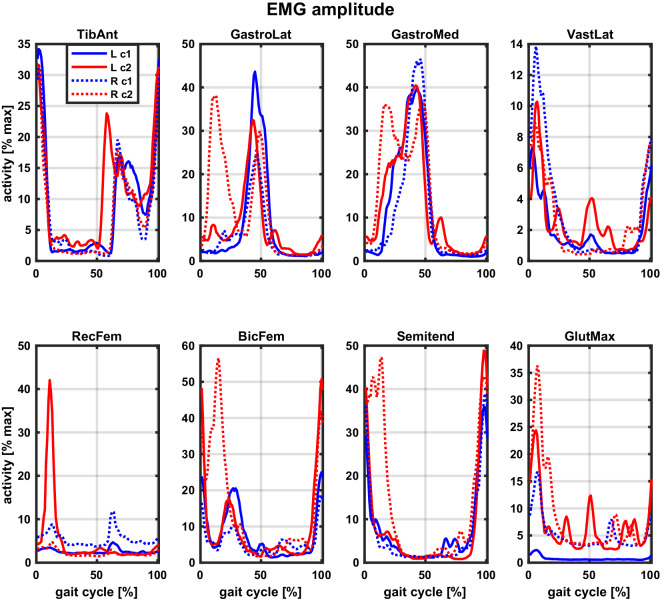
Example EMG amplitude across conditions (taken from participant S1). Each subplot corresponds to a different muscle. Note different y-axis limits. The free and constrained conditions are colour-coded, with blue corresponding to free (c1) and red to constrained (c2) condition. Full lines correspond to the left leg (L) and dotted lines to the right leg (R). Each muscle’s EMG amplitude is normalised using maximum amplitude measured across walking, stair ascending, and sit-to-stand activity in the corresponding session (in this case, free walking in Session3 and constrained walking in Session2; the actitivies with the highest amplitude can be found in Supplementary File, Table S2). Data are taken from walking at 1.1 m/s and the preferred cadence. NOTE: Reader is referred to online version of this manuscript for colour-coding.

**Fig. 11:**
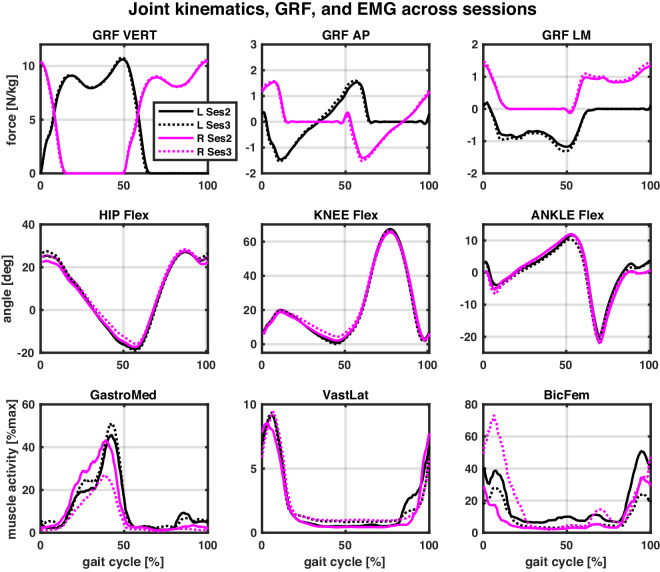
Example joint kinematics, GRF, and muscle activity across two sessions (taken from participant S13). The top row shows GRF in all three planes (normalised to the person’s mass), the middle row joint kinematics in the sagittal plane, and the bottom row muscle activity of three muscles (limited to three for clarity of presentation). All data were measured while walking at the preferred speed at the start of Session 2 and Session 3 (i.e., *PrefStart* field in the provided dataset). The left and right legs are colour-coded, with black corresponding to the left (L) and magenta to the right (R) leg. Full lines correspond to Session 2 (Ses2), and dashed lines to Session 3 (Ses3). EMG data were normalised per muscle by the peak measured muscle activation from the pool of three activities (walking, stair negotiations, sit-to-stand). In this case, the same activities were used on both legs, as follows (Sess2/Sess3): Walking/Stairs for GastroMed, STS/Stairs for VastLat, and Stairs/Walk for BicFem. NOTE: Reader is referred to online version of this manuscript for colour-coding.

**Fig. 12:**
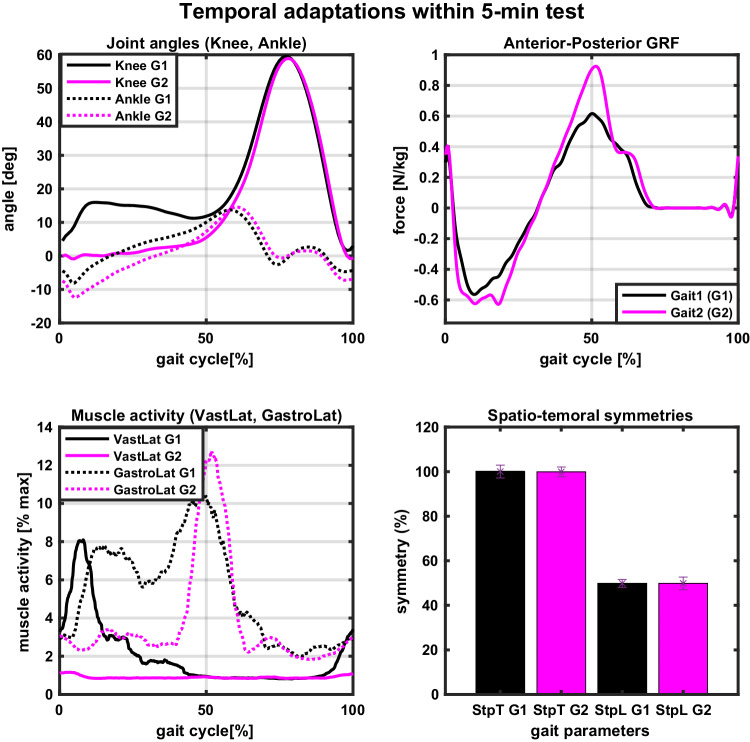
Example temporal adaptations within a single 5-minute test (taken from participant S15, test T14 - walking free (c1) at 0.4 m/s and +10% cadence). The participant underwent two distinct gait patterns, called *Gait1* (black line/bar) and *Gait2* (magenta line/bar). Gait pattern *Gait1* was taken from cycles 1-25, and gait pattern *Gait2* from cycles 50-75. All trajectories and point-values (symmetries) are averages over the respective 25 cycles. Trajectories are taken from the left leg. Top left: Knee (full line) and ankle (dotted line) joint angles in the sagittal plane during two gait patterns. Top right: GRF in anterior-posterior direction for two gait patterns. Bottom left: Muscle activity of VastLat (full line) and GastroLat (dotted line) during two gait patterns. Bottom right: Step time symmetry *StpT* (black bar) is calculated as *Left Step Time / Right Step Time*, and step length symmetry *StpL* (magenta bar) is calculated as *Left Step Length / (Left Step Length + Right Step Length)* for both gaits, with 100% and 50% denoting perfect symmetry respectively. In particular, *StpT G1* = 100.04, *StpT G2* = 99.91, *StpL G1* = 49.86, and *StpL G2* = 48.76. NOTE: Reader is referred to online version of this manuscript for colour-coding.

The collected data was validated to confirm consistency within and across participants, as well as in comparison to available datasets, providing confidence in its physiological accuracy and relevance. Representative trajectories are visualised by taking different participant’s data from a single session, as follows: ground reaction forces of S2 in Fig. 7; joint kinematics data of S8 in Fig. 8; metabolic activity of S17 in Fig. 9, and electrical muscle activity of S1 in Fig. 10. Further dataset validation and a demonstration of the sort of analyses enabled by our dataset is provided by comparing joint kinematics, GRF, and EMG data from S13 across the two sessions (during a 6-minute *PrefStart* walking trial) in Fig. 11 and by visualising temporal adaptations during a single 5-minute test from S15 in Fig. 12. All data are segmented between the subsequent heel strikes of the same leg; for visualisation purposes, GRF data of both legs are plotted between left heel strikes; on the other hand, joint angles and muscle activity are plotted between ipsilateral heel strikes (left side between left, and right side between right heel strikes). All trajectories are averaged over the last 20% of cycles (approximately the last minute of walking in a 5-minute test), and data are taken from the tests with the preferred cadence.

**Fig. 13:**
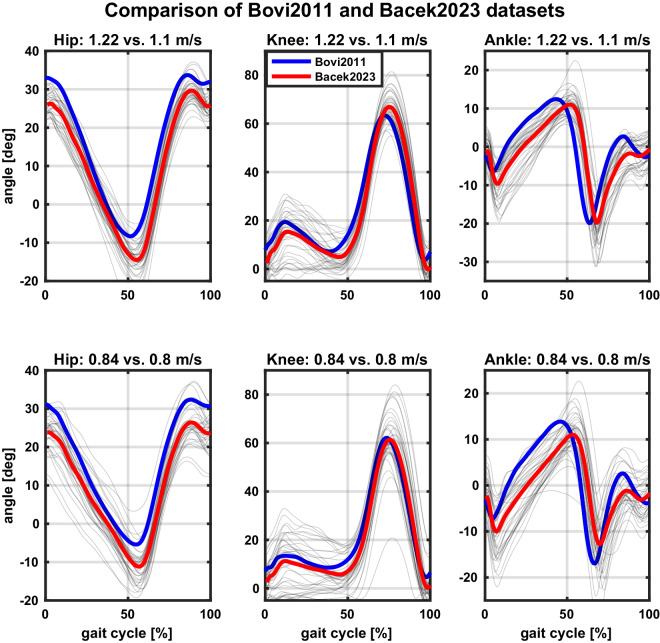
Comparison of our dataset to that of Bovi *et al.* The two rows correspond to two different walking speeds similar but not equal across the two datasets) and columns to the three lower limb joints in the sagittal plane. The blue (Bovi *et al*.) and red (Bacek *et al*.) trajectories are mean across all participants and both legs in the two datasets and grey trajectories are actual joint angles from our dataset. Data come from walking at preferred cadence. Note that the comparison is made between different speeds: 1.22 vs. 1.1 m/s in the top row, and 0.84 vs. 0.8 m/s in the bottom row, Bovi *et al*. vs. Bacek *et al*. datasets, respectively. NOTE: Reader is referred to online version of this manuscript for colour-coding.

To assess the validity of the presented dataset, a comparative analysis is provided with two publicly available datasets that have similar walking speed conditions as our dataset. The first publicly available dataset used in the comparison is Bovi *et al*.^10^, which presents a multi-task analysis of human walking (and stair negotiation) in 20 healthy adults whose age, height, and weight distributions include the ones found in our sample (in the Bovi dataset, the participants were 43 ± 15 years of age, with average body mass 68.5 ± 15.8 kg and height 1.71 ± 0.1 m). The comparison is limited, however, due to the differences in the ways data were collected. Bovi *et al*. collected their data by asking participants to walk over a walkway as opposed to a treadmill, which limits their number of gait cycles and does not account for familiarisation taking place when walking over longer periods. Furthermore, the participants in Bovi *et al*. first walked at their preferred speed, which was then progressively increased and decreased without any precise indications of the desired gait speed. During the analysis, walking speeds were *a-posteriori* categorised into *very slow*, *slow*, *medium*, and *fast* walking defined by the ratio of the freely chosen speed and participant’s height. To allow comparison between the two datasets, we multiplied the average participant’s height by the average gait speed in each of the four categories and took data from two speed categories that most closely resemble gait speeds in our study. These included 1.22 m/s and 0.84 m/s, which are compared to our 1.1 m/s and 0.8 m/s trials (see Fig. 13).

**Fig. 14:**
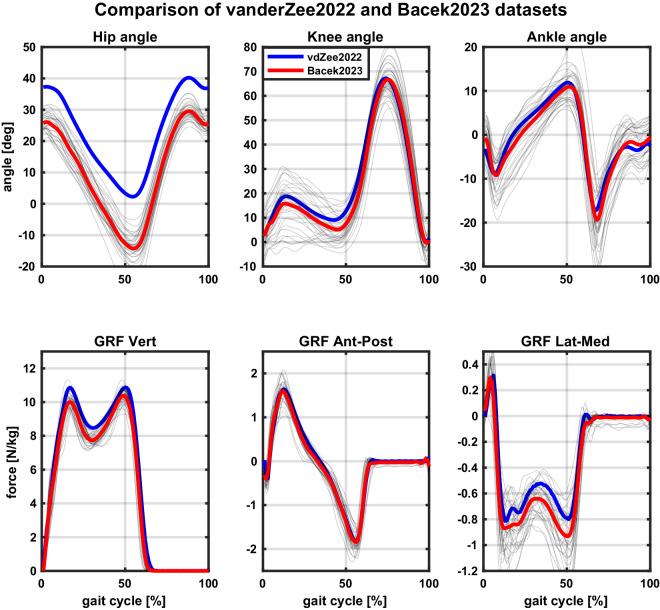
Comparison of our dataset to that of van der Zee *et al.* The top row shows a comparison of the three lower limb joint angles in the sagittal plane and the bottom of ground reaction forces (GRF) in all three directions. The blue (van der Zee *et al*.) and red (Bacek *et al*.) trajectories are mean across all participants and both legs in the two datasets and grey trajectories are actual joint angles from our dataset. The comparison is made between data at the same speed of 1.1 m/s and preferred gait parameters (step length and cadence) in both datasets. NOTE: Reader is referred to online version of this manuscript for colour-coding.

The second publicly available dataset used in the comparison is van der Zee *et al*.^72^, which presents data of ten healthy adults walking at a range of speeds (0.7-2.0 m/s) in a lab. With a mean age of 24 ± 3 years, an average body mass of 73.5 ± 15 kg, and an average height of 1.76 ± 0.11 m, the participants in van der Zee *et al*. study are similar in size albeit a bit younger compared to participants in our study. Different from comparing our dataset to the Bovi *et al*. dataset, though, the comparison to van der Zee *et al*. dataset is more direct due to several similarities in data collection. These include subjects walking on a dual-belt instrumented treadmill at their preferred gait and 1.1 m/s. For this reason, it was possible to compare speed-matched joint kinematics and GRF, which are given in Fig. 14. A notable difference between the two datasets are shorter tests in the van der Zee *et al*. dataset, where participants were asked to walk only 60 seconds per condition compared to 300 seconds in our dataset.

## Usage Notes

During data collection and exporting from the Vicon Nexus software, we’ve encountered several issues that are reflected in the provided datasets. To avoid these interfering with data analysis and explain occasional unexpected results, a summary of the kinematics and kinetics data features and issues is summarised in Supplementary File, Table S3 and ground reaction force issues in Table S4. Generally, the issues can be separated into test-related and export-related. The test-related issues refer to instances when a test had to be briefly stopped due to, e.g., a loose marker, which resulted in a short period of unusable data (the test recording was not stopped when such interruption happened). On the other hand, export-related issues refer to an atypical sampling frequency of the data exported from Nexus, such as ground reaction forces exported at 2 kHz instead of 1 kHz. The corrections made to the data, such as removing unusable data of a person standing on the treadmill due to marker fixture or downsampling GRF data to 1 kHz, are only done in the MAT files. The C3D files contain the raw data as it was collected/exported, with no corrections whatsoever.

### Limitations

The study is designed with a focus on individual adaptations rather than trends or averages across groups. This is seen in the same group of participants being both the *control* and the *intervention* group, resulting in 30 different conditions for each participant, sufficient to investigate individual adaptations. At the same time, a small sample size (N=21) requires a cautious approach when the focus shifts away from individuals, and data are used to find trends and averages in the sample and apply the findings to the population the sample is taken from. Further to the sample characteristics, the study recruited predominantly male participants despite the efforts to make the gender distribution as equal as possible.

Studies that analyse human gait in a controlled lab environment use both overground and treadmill walking protocols, albeit more often the former^13^. Despite some researchers calling for a cautious translation of results between the two, no clear and uniform differences seem to exist between the two protocols^40^. Where our treadmill-based dataset does fall short is in addressing spontaneous changes in walking speed as documented by Ojeda *et al*.^73^. Finally, due to several issues with the PNOE metabolic cost analyser we experienced with some of the subjects, the metabolic cost measurement is missing in 36 out of 330 5-minute tests collected (about 11%). However, statistical and computational modelling tools exist in the literature that can be used to estimate the missing test values using the available MC test values. Notably, some statistical analyses can handle the amount of missing data in our dataset without the loss of statistical power.

The EMG amplitude is normalised to the peak EMG signal measured during three everyday activities – walking, sit-to-stand, and stair climbing. As highlighted by Besomi *et al*.^70^, this approach limits the normalised data analysis to the following contexts:

- amplitude comparison within a person and muscle, within a session;
- amplitude comparison within a person and muscle, across sessions (with caution);
- comparison of shape/activation patterns (timing of bursts, timing of peaks, period of inactivity).

A direct amplitude comparison between muscles is not recommended with this EMG amplitude normalisation method since the comparison is done to the muscles peak activation within a given activity and not its maximum contractive capacity.

### Supplementary information

Supplementary Info

## Data Availability

A file containing multiple Matlab® scripts is available on figshare^49^ as a separate .zip file with the suffix *Other*. These scripts allow simple analysis and visualisation of ground reaction forces and joint angles (*MAIN_KineticsKinematics.m*), metabolic cost and cost of transport (*MAIN_MetabolicCost.m*), and muscle activation (*MAIN_Emg.m*). The same code can also be found on the project’s GitHub website, accompanied by the instructions on how to use the code and description of each of the three main and several auxiliary scripts/functions.
