# Supplementary material for: A biomechanics and energetics dataset of neurotypical adults walking with and without kinematic constraints
Source: Sci Data. 2024 Jun 18;11:646. doi: 10.1038/s41597-024-03444-4 (PMC11189391; doi:10.1038/s41597-024-03444-4)
Supplement: Supplementary file 1 — Supplementary Info [file 41597_2024_3444_MOESM1_ESM.pdf]

Supplementary File

A biomechanics and energetics dataset of neurotypical adults walking with and without kinematic constraints

Tomislav Baček, Mingrui Sun, Hengchang Liu, Zhongxiang Chen, Chris Manzie,
Etienne Burdet, Dana Kulić, Denny Oetomo, Ying Tan

Table of Contents

EMG delay correction 2

EMG data issues/quality 3

EMG normalisation activities 6

Kinematics and kinetics data issues/quality 7

Ground reaction force issues 9

EMG delay correction

In subjects S1-12, EMG data and Vicon data (markers and ground reaction forces, GRFs) were not synchronised during data collection due to the missing hardware synchronisation trigger. The delay for these subjects is corrected in post-processing, using a marker and an EMG sensor from the right shank due to their close proximity. The standing assumption here is that accelerations in corresponding axes in both the marker and the IMU are very similar and can be matched by shifting one signal. A shift corresponds to a time delay, subsequently used to synchronise ground reaction forces and joint angles (from Vicon markers) with muscle activations (from the Trigno EMG sensors). The assumption is validated using trigger-synchronised data from subjects S13-21.

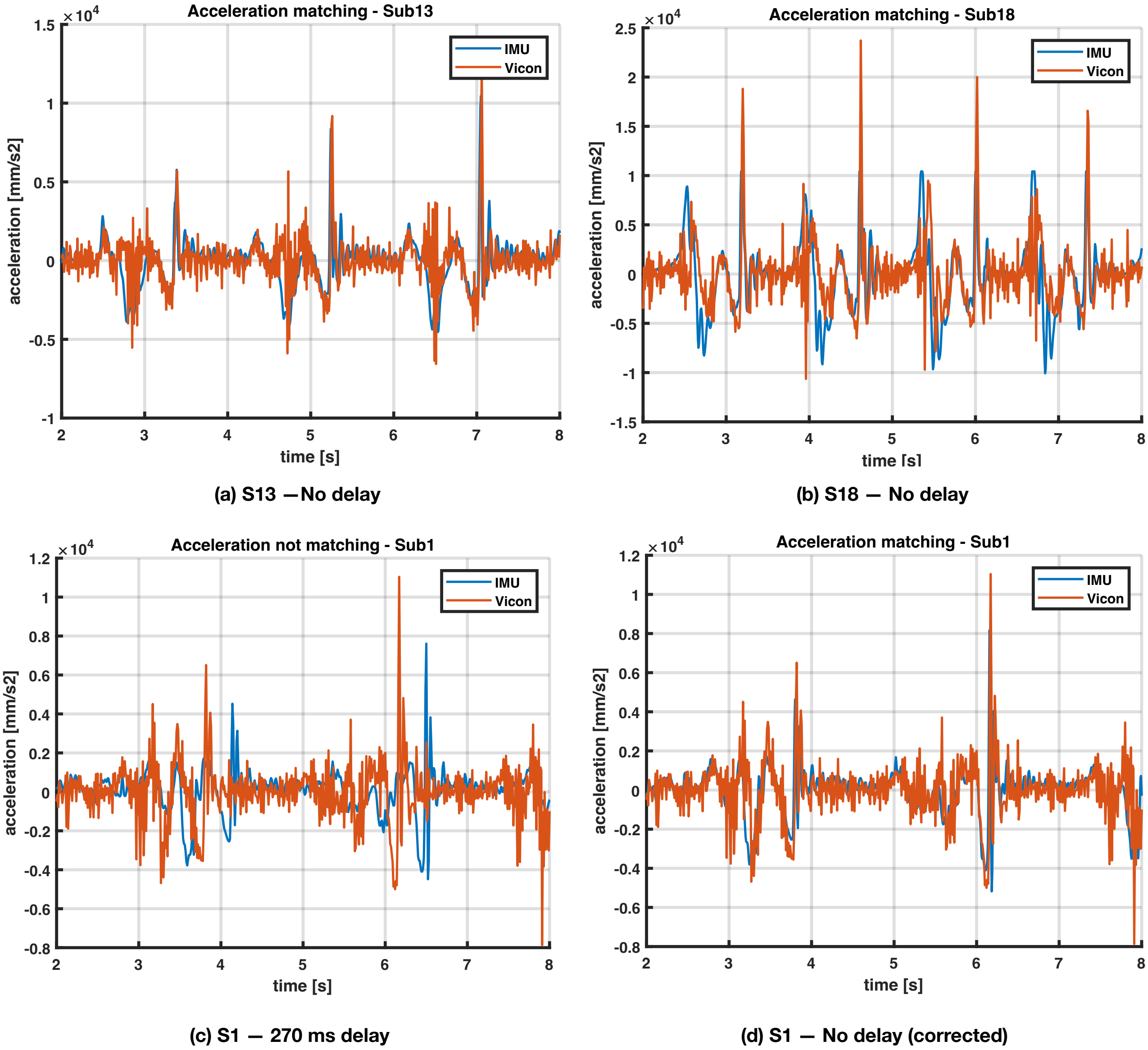

*Figure S1: Acceleration data from IMU (Delsys Trigno) and Vicon marker sensors used in correcting EMG signal delay. A comparison and correction are made using an acceleration signal from the Right Tibialis Anterior (RTibAnt) sensor and auxiliary Right Tibia marker (RTIBExt1), both in their Z-directions. For calculations, the IMU signal is downsampled to 100 Hz frequency (Vicon sampling) from its original 2000 Hz. The cross-correlation algorithm compares the two signals and outputs a delay in [ms] for achieving the best possible match. Big spikes in the marker signal (orange) correspond to the heel strike event. (**a**) and (**b**) show data from S13 and S18, respectively, collected with trigger-based data synchronisation between EMG and Vicon signals. (**c**) and (**d**) show original and corrected, respectively, signals from Sub1. The former demonstrates an offset (delay) in Delsys Trigno sensors and the latter an excellent match once the delay is corrected.*

EMG data issues/quality

An overview of muscle activation data quality. The data are organised per person per session. Subjects S7 and S12 did not go through Session3, which is why NA (not applicable) is used. In general, a good signal is in range between 3 microV (noise) and 500 microV RMS amplitude. **PS** = preferred walk start; **PE** = preferred walk end; **Silent muscles** = SNR < 6 dB (signal:noise RMS amplitude 2:1); **Low activity** = amplitude RMS < 10 microV and SNR > 6; **High activity** = amplitude RMS > 500 microV; **Abnormal** = sensor saturating; **Intermittent** = signal switching between noise and activity; **Poor signal** = higher-than-normal noise levels with no clear activity. NOTE: only Abnormal and Poor signal indicate unusable data. Negative delay means the EMG (IMU) signal was late compared to the Vicon marker signal.

Table S1. An overview of issues in EMG data encountered during data collection.

	Session 2	Session 3
**S1**	**Silent muscles**: RRecFem (T11-15, T21), LRecFem (T11-15); **Low activity**: RRecFem (T16, T18, T21-23), LRecFem (T16, T18, T21-24), RBicFem (T11-15), RGlutMax (T13), LGlutMax (T13, T15); **Delay correction**: -270 ms across the session; **Other**: LBicFem increasing throughout test (PE).	**Silent muscles**: LRecFem (T26-30), RRecFem (T29-30); **Low activity**: LBicFem (T26-30), RRecFem (T6-10, T27-28), RBicFem (T27-28, 30), LRecFem (T6-10); **Delay correction**: 0 ms; **Other**: LGastroLat and LGastroMed fell off after Bout1 (T1-5) and were re-attached using skin marks made by electrodes without re-normalisation.
**S2**	**Silent muscles**: LGlutMax (T26-30)< RGlutMax (T26-30); **Low activity**: LBicFem (T29), LRecFem (T26-28, T30), RRecFem (T26-27); **High activity**: LGastroLat (T21-25, T28, T30, PE); **Delay correction**: -260 ms across the session.	**Silent muscles**: RGlutMax(T6-15), LGlutMax (T12); **Low activity**: LBicFem (T11-12, T14-15), LRecFem (T11-12, T14-15), LGlutMax (T14), RRecFem (T15); **Delay correction**: -270 ms across the session.
**S3**	**Silent muscles:** LVastLat (T6-9, T27-28), LRecFem (T6-9), RRecFem (T7), LGlutMax (T26-30), RGlutMax (T26, T28, T30; **Low activity**: RRecFem (T6, T8, T18, T27), RGlutmax (T6, T27, T29), LGlutMax (T10), RBicFem (T26, T28-29), LBicFem high (T27-28, T29 end), LVastLat (T26, T29); **High activity**: LGastroLat (T16, T19, PE end), LGastroMed (T16, T19, PE start), RSemitend (T17-20); **Abnormal**: LTibAnt (T19 mid), LBicFem (PE start); **Intermittent**: RVastLat (T7-8), LRecFem (T26-29); **Delay correction**: NA.	**Silent muscles**: LVastLat (T13-15, T21, T23), LRecFem (T11-15, T21, T23), LGlutMax (T11-15), RRecFem (T14, T21, T23), RGlutMax (T15); **Low activity:** RBicFem (T11-15), RGlutMax (T11-15, T24-25), RRecFem (T11-13, T15, T22), LBicFem (T11-13, T15), RVastLat (T21-22), LVastLat (T22), LGlutMax (T22, T24), LRecFem (T24); **Intermittent**: LVastLat (T11-12), RVastLat (T23); **Delay compensation:** NA (except PS: -1030 ms).
**S4**	**Silent muscles**: LVastLat (PS, T1-2, T4, T21, T23, T26-30, PE), LRecFem (T23, T26-30), RVastLat (PS, T23, T27-28, PE), LGlutMax (T26-30), RRecFem (T27-28), RGlutMax (PS, PE); **Low activity**: RRecFem (T1-3, T21-24, T29-30), LVastLat (T5, T22, T24-25), LRecFem (T1, T3, T21-22, T24-25, PE), LGlutMax (PS, T1-3, T22, T24, T26, PE), RVastLat low (T1-5, T21), RGlutMax (T1-5, T26-28); **High activity**: LGastroLat (T1-2, T21-22, T21–25); **Abnormal**: LGastroLat (T3-5); **Intermittent**: LGastroMed (T30); **Delay correction:** [T1… T5] = - [30 70 140 360 270] ms; [PS PE] = - [360 30] ms; -1180 ms the rest of the session.	**Silent muscles:** LVastLat (PS, T6-11-17, T19-20, PE), LGLutMax (PS, T6-15), RVastLat (PS, T6-7, T9, T11-15), RGLutMax (PS, T6-15, PE), LRecFem (T6-7), RRecFem (T6); **Low activity**: RRecFem (T11-12, T17), LRecFem (T11-12, PE), LGastroLat (T15), LRecFem (T17-18, T20), RGlutMax (T17), RVastLat (T8), LGLtuMax (PE); **Delay correction:** -1060 ms across the session.
**S5**	**Silent muscles**: LGlutMax (T11); **Low activity**: RGlutMax (T11-13), LGlutMax (T12-13), RRecFem (T1, T21); **High activity**: LTibAnt (T3 end, T21, T23 start, PE end); **Intermittent**: RVastLat (T11-15), RBicFem (T11-12), RRecFem (T11-15), RSemitend (T12), LVastLAt (T11-15), LRecFem (T11-15), LBicFem (T12-13), LTibAnt (T1); **Abnormal**: LVastLat (T1-15, PE); **Delay correction**: -270 ms across the session.	**Silent muscles**: LRecFem (T7); **Low activity**: LRecFem (T6, T8), LGlutMax (T26-27, T29-30), RRecFem (T28); **Intermittent**: LRecFem (T26-30), LSemitend (T26-27), RVAstLat (T26-29); **Delay correction**: -260 ms across the session (excluding PS); **Other**: LVastLat strange spikes (T6-8, T26-30).

	Session 2	Session 3
**S6**	**Silent muscles**: LRecFem (T15), LGlutMax (T11-15), RRecFem (T15); **Low activity**: RVastLat (T11, T13-15), RRecFem (T11-14, T24), LVastLat (T11-15, T21-25), LRecFem (T11-T14), LBicFem (T15, T24), LGlutMax (T22, T24-25); **High activity**: LGastroMed (T1-4, T21, T24); **Abnormal**: LGAstroMed (T5, PE); **Delay correction**: -270 ms across the session.	**Silent muscles**: None; **Low activity:** RRecFem low (T6-9, T19, T27), RGlutMax low (T7-9, T19-20), LVastLat low (T19-20, T26, T28-30), LGlutMax low (T19), RBicFem low (T7-9, T30), LBicFem low (T8, T10, T27-30), LRecFem (T27-28), RVastLat (T27-28), LSemitend (T29); **Abnormal**: RVastLat (PS); **Delay compensation**: -250 ms across the session.
**S7**	**Silent muscles**: RVastLat (T7, T11-15), LVastLat (T7, T11-15), RRecFem (T12-14); **Low activity**: RGlutMax (T7, T11, T15), LVastLat (T6, T8-9), LRecFem (T11); **Delay correction**: [PS T1 T2 … T15 PE] = [0 140 100 80 0 -70 190 220 90 30 0 320 280 220 100 10 0] ms.	NA
**S8**	**Silent muscles**: RGlutMax (T11-13), RRecFem (T6, T11), RVastLat (T6, T11); **Low activity**: RRecFem (T7, T11-14), LGlutMax (T11-14, T20), RGlutMax (T14-15), RBicFem (T15), LRecFem (T11, T13), LVastLat (T20), RVastLat (T7); **High activity**: RBicFem (T20 end); **Intermittent**: RVastLat (T7-8, T11-14), LVastLat (T6, T11, T13); **Delay correction**: -280 ms across the session.	**Silent muscles**: LGlutMax (T26-29); **Low activity**: RRecFem (T21, T23, T26-27, T30), LRecFem (T26-27, T29-30), LVastLat (T26-27, T29), LGlutMax (T2, T23), RGlutMax (T2); **Intermittent**: RVstLat (T21-23, T26-27); **Delay correction**: -1160 ms across the session – for reasons of long delay, all tests have one cycle less in EMG than in kinematics/kinetics.
**S9**	**Silent muscles**: LGlutMax (T11-15), LVastLat (PS, T4, T12-13, PE), LRecFem (T12-13), RVastLat (T14-15), RRecFem (T14-15); **Low activity**: RRecFem (T11-13), RGlutMax (T11-13), RVastLat (T11, T13) LRecFem (T11, T23), LVastLat (T11), LGlutMax (T23-24); **Intermittent**: LVastLat (T14-15), LRecFem (T14-15), RVastLat (T23); **Poor signal**: RGlutMax (T1-3, T21-22, T25), RRecFem (T1-3, T21-22, T25), RVastLat (T1-3, T21-22, T25), LGlutMax (T1-3, T21-22, T25), LRecFem (T1-3, T21-22, T25), LVastLat (T1-3, T21-22, T25); **Delay correction**: -280 ms across the session.	**Silent muscles**: LVastLat (PS, T6-10, T26-29, PE), LRecFem (T7-8), RVastLat (T7, T9-10, T27-28), RRecFem (T6, T8-9, T26-29), LGlutMax (T28-30); **Low activity**: LGlutMax (T16, T18, T20, T26), RRecFem (T7, T16, T18, T20), RGlutMax (T16, T18, T20, T27), RVastLat (T8, T20), LRecFem (T6, T26-30); **High activity**: LBicFem (T8, T28-30); **Intermittent**: RVastLat (T26, T28); **Abnormal:** LBicFem (PE); **Delay correction**: -320 ms in Bout3 (T26-30) and PE, no correction otherwise.
**S10**	**Silent muscles**: LVastLat (T23-35), LGlutMax (T21-25), LRecFem (T26-30), LVastLat (PS, T26-29), LGlutMax (T26-30); **Low activity**: LRecFem (PS, T25), LGlutMax (T3-5, PE), RGlutMax (T3-5, T27-28, T30, PE), LGastroLat (T29); **High activity**: RVastLat (PS, PE); **Abnormal**: RGLutMax (PS); **Delay correction**: -1020 ms (PS, T1-5, T21-25), and -260 (T26-30, PE) – for reasons of long delay, PS, T1-5, and T21-25 have one cycles less in EMG than in kinematics/ kinetics.	**Silent muscles**: LVastLat (PS, T6-15, PE), LRecFem (PS, T6-18, PE), LGlutMax (T11-15), RVastLat (T13-15), RRecFem (T12-15), RGlutMax (T12-15); **Low activity**: RRecFem (T11), RGlutMax (T7-11, PE), LGastroLat (T11, T14, T20), LGastroMed (T20), LVastLat (T20), LGlutMax (T19-20), LRecFem (T19-20); **Intermittent:** RVastLat (T11); **Delay correction**: -280 ms across the session.
**S11**	**Silent muscles**: LGlutMax (PS, T1-10, T26-30, PE), RGlutMax (T7, T26-30); **Low activity**: LRecFem (T7, T26-27), RRecFem (T27), RGlutMax (T4), LBicFem (T6, T8); **Delay correction**: -270 ms across the session.	**Silent muscles**: LGlutMax (T11-15), RGlutMax (PS, T11-15, T21-23, PE), LBicFem (T13, T15); **Low activity**: RRecFem (T11-13, T15-18, T21), RGlutMax (T16-18, T24-25), LGlutMax (T16-18, T23), LRecFem (T11-14, T18, T21), LVastLat (T12), LBicFem (T11-15, T21, T23); **Delay correction**: 270 ms across the session.
**S12**	**Silent muscles**: LGlutMax (T11-15), LVastLat (T13-14), RGlutMax (T13), RRecFem (T13); **Low activity**: RRecFem (T11-12, T14-15), RGlutMax (T11-12), RVastLat (T13 end, T23 first half), LGlutMax (T22); LRecFem (T13), **High activity**: LGastroLat (T14), RGastroLat (T11, T13), LSemitend (T22 first half, T23, T25); **Intermittent:** LGastroLat (T25), LGlutMax (PS); **Abnormal**: RGastroLat (PS); **Delay correction**: 290 ms across the session; **Other**: PE missing; T4, T5 not segmented;	NA

	Session 2	Session 3
**S13**	**Silent muscles**: LGlutMax (PS, T23-24, T26-30, PE), RGlutmax (T26-30), RBicFem (T26, T28, T30), LRecFem (T27-28); **Low activity**: LGlutMax (T4-5, T21-22), RBicFem (T27, T29), LGastroLat (T30), LVastLat (T27)**.**	**Silent muscles**: LGlutMax (PS, PE, T7-9, T11-13, T15), RGlutMax (T11-15); **Low activity**: RGastroLat (T15), LGastroLat (T12-15), LGlutMax (T10, T19-20), RGlutMax (T6, T8-9, T19); **High activity**: RBicFem (T6-7, PS, PE).
**S14**	**Silent muscles**: LRecFem (T11-15), RRecFem (T13-14), RBicFem (T15); **Low activity**: RRecFem (T6-8, T10, T12-20), RBicFem (T12-14), LVastLat (T8-9, T14-20), LRecFem (T8-9, T16-20), LGlutMax (T12-13), RGlutMax (T11-13); **Abnormal**: LGlutMax (T10, T14, T17-20, PS, PE), RGlutMax (T6-7, T16, PS); **Other**: LGlutMax and RGlutMax only noise (T6-7, T9-10).	**Silent muscles**: LGlutMax (T26-30), RGlutMax (T26-30). **Low activity**: LRecFem (T21, T26-30), LBicFem (T28, T30), LGlutMax (T21-22).
**S15**	**Silent muscles**: RGlutMax (T6-15, PE), LGlutMax (T12-15), RRecFem (T12, T14); **Low activity**: RRecFem (T6-8, T11, T13, T15), LRecFem (T9, T11-12, T15), RBicFem (T6), LGlutMax (T6); **High activity**: RVastLat (T6-11, T13-14, PS, PE); **Abnormal**: RVastLat (T16, T18-20)	**Silent muscles**: NA; **Low activity:** LRecFem (T24, T26-30), RRecFem low (T26-30); **Steady amplitude increase**: RGastroLat (PS, T1, T24, PE), LGastroLat (PE); **Other**: LGlutMax and RGlutMax not available through the session.
**S16**	**Silent muscles**: LRecFem (T26-27, T30), LGlutMax (T26-28, T30); **Low activity**: LGlutMax (T3, T6, T8), RGlutMax (T6-10), LGastroLat (T26-30), LVastLat (T26-30); **High activity**: RTibAnt (T5), LGastroLat (PE), LVastLat (PE); **Abnormal**: RTibAnt (T1-3, T4 start, T6-10, T26-30, PS, PE), RVastlat (T1-3, T4 start, T5 end, T6-10, T26 end, T27-30, PS, PE)	**Silent muscles**: LVastLat (T11-15), LGlutMax (T11-15), LRecFem (T11-13), RRecFem (T11-13, T15); **Low activity**: LRecFem (T17, T20), LGlutMax (T20-21, T22, T24), RRecFem (T17, T21), RGlutMax (T15); **High activity**: LRecFem (T16, T19), LBicFem (T16, T18-19), RVastLAt (T18) LGastroMed (T19), LSemitend (PE); **Abnormal**: LVastLat (T16, T18-19), RVastLat (T16, PS)
**S17**	**Silent muscles**: RGlutMax (T11-15, T17, T19-20, T21-25, PS, PE), LGlutMax (T11-15, T22-25); **Low activity**: RRecFem (T16, T18, T20), LGlutMax (T16, T18, T20-21, PE), RGlutMax (T16), RBicFem (T11-13), LRecFem (T11-13, T21, T23-25); **High activity**: LGastroLat (T16, T18, T20-21, T23-25), LGastroMed (T18).	**Silent muscles**: RGlutMax (T1-10, T26-30, PS, PE), LGlutMax (T9), RBicFem (T28-29), LRecFem (T26-27); **Low activity**: LRecFem (T6-9, T28-29), LBicFem (T27-29), LGlutMax (T1-6, T8, T10, T26-30, PE), RBicFem (T26-27, T30); **High activity**: LGastroLat (T10)
**S18**	**Silent muscles**: LRecFem (T11, T13-14), RVastLat (T13), RRecFem (T11-15), RGlutMax (T11-15); **Low activity**: LRecFem (T21-24), RRecFem (T11-18, T24), LBicFem (T21, T23), **Intermittent**: RVastLat (T11-13).	**Silent muscles**: LRecFem (T6-10, T26-30), LGlutMax (T6-8, T26-30), RRecFem (T6-10, T26-30); **Low activity**: LGlutMax (T5), RRecFem (T1-3), RGlutMax (T1-2, T27, T30), LRecFem (T1-2); **High activity**: LGastroLat (T6-10, PE); **Abnormal**: LGastoMed (T26, T28); **Intermittent**: RVastLat (T26-T29).
**S19**	**Silent muscles**: RRecFem (T11, T14-15); **Low activity**: RGlutMax (T11-15), RBicFem (T12, T15), RRecFem (T12), LRecFem (T11, T14-15, T20), LGlutMax (T11, T15); **High activity**: RGlutMax (T6-10, T19), LGastroMed (T17), LGastroLat (T16 end, T17, T20 end); **Abnormal**: LGastroMed (T20), RVastLat (T6-10), RGlutMax (PE); **Other**: LGlutMax strange (PS).	**Silent muscles**: LRecFem (T27-30), LGlutMax (T26-30); **Low activity**: LGlutMax (T4-5, T23, T25), RGlutMax (T23), RBicFem (T26, T28-30), LRecFem (T26); **High activity**: LGastroMed (T2, T4); **Other**: LGastroLat and LGastroMed touching cuff (T23-35).
**S20**	**Silent muscles**: RGlutMax (T26-30); **Low activity**: RRecFem (T19, T26-30), LRecFem (T26, T28-29), LBicFem (T26, T29), LGlutMax (T28), RGlutMax (T7, T9-10).	**Silent muscles**: LGlutMax (T11-15), RGlutMax (T11-15); **Low activity**: LGlutMax (T1, T3, T21-23), RRecFem (T12-13), LRecFem (T12-13); **Intermittent**: RVastLat (T11-15), LBicFem (T12-13), LSemitend (T12-13).
**S21**	**Silent muscles**: RVastLat (T6-9, T26-28, T30, PE), RRecFem (T6-7), LRecFem (T6-7); **Low activity**: RRecFem (T1-2, T8), LGAstroLat (T30), LRecFem (T1-2, T26-30); **Other**: LGastroMed touching orthosis (T27, T29).	**Silent muscles**: RRecFem (T11-15, T21-23), RVastLat (T11-15, T24), RGlutMax (T11-15), LGlutMax (T11, T13-15), LRecFem (T13, T21-23), LVastLat (T13-15, PS, PE); **Low activity**: RRecFem (T16), RBicFem (T11, T14), LRecFem (T11-12, T15, T24-25), LVastLat (PE).

EMG normalisation activities

Table S2 below gives an overview of activities (walking, stair negotiation, sit-to-stand) used in muscle activity normalisation as presented in the paper. Top and bottom row per subject correspond to Sess2/Sess3.

Table S2. Activities used in muscle activity normalisation (STS = sit-to-stand; Walk = preferred walking)

	LEFT								RIGHT							
Sub/ Musc	Tib Ant	Gastr oLat	Gastr oMed	Rec Fem	Vast Lat	Bic Fem	Semit end	Glut Max	Tib Ant	Gastr oLat	Gastr oMed	Rec Fem	Vast Lat	Bic Fem	Semit end	Glut Max
S1	STS	Stairs	Stairs	STS	STS	Walk	Walk	Stairs	STS	Stairs	Stairs	Stairs	Stairs	Stairs	Walk	Stairs
	STS	Stairs	Stairs	Stairs	STS	Stairs	Walk	Stairs	STS	Stairs	Walk	Stairs	STS	Stairs	Walk	Stairs
S2	STS	Stairs	Stairs	STS	STS	Stairs	Walk	Stairs	STS	Stairs	Stairs	STS	STS	Walk	Walk	Stairs
	STS	Walk	Walk	STS	STS	Walk	Walk	Stairs	STS	Stairs	Stairs	STS	STS	Stairs	Stairs	STS
S3	STS	Stairs	Stairs	Stairs	STS	Stairs	STS	STS	STS	STS	Stairs	Stairs	STS	STS	Walk	STS
	STS	Walk	Stairs	STS	STS	Stairs	Walk	STS	STS	Stairs	Stairs	Stairs	STS	Stairs	Walk	STS
S4	STS	Stairs	Stairs	Stairs	STS	Walk	Walk	STS	STS	Stairs	Walk	STS	STS	Walk	STS	STS
	STS	Walk	Stairs	STS	STS	Stairs	Walk	STS	STS	Stairs	Stairs	Stairs	STS	Stairs	Walk	STS
S5	Walk	Stairs	Stairs	STS	Stairs	Walk	Stairs	Stairs	STS	Stairs	Stairs	STS	STS	STS	Stairs	STS
	STS	Stairs	Stairs	Stairs	STS	Walk	Stairs	Stairs	STS	Stairs	Stairs	STS	STS	Walk	STS	Walk
S6	STS	Stairs	Stairs	STS	STS	Stairs	Stairs	Stairs	STS	Stairs	Walk	STS	Stairs	Stairs	Stairs	Stairs
	STS	Stairs	Stairs	STS	Walk	Stairs	Stairs	Stairs	STS	Stairs	Stairs	STS	Stairs	Stairs	Stairs	Stairs
S7	STS	Stairs	Stairs	STS	STS	Stairs	Stairs	Walk	STS	Stairs	Walk	Stairs	Stairs	Walk	Walk	Stairs
	–	–	–	–	–	–	–	–	–	–	–	–	–	–	–	–
S8	STS	Stairs	Walk	STS	Stairs	Walk	Stairs	Stairs	STS	Stairs	Stairs	Stairs	Stairs	Walk	Walk	Stairs
	STS	Stairs	Stairs	Stairs	STS	Walk	Walk	Stairs	STS	Stairs	Stairs	Stairs	Stairs	Stairs	Stairs	STS
S9	STS	Stairs	Stairs	Stairs	Stairs	Stairs	Stairs	Stairs	STS	Stairs	Stairs	Stairs	Stairs	Stairs	Stairs	Stairs
	STS	Stairs	Stairs	Stairs	Stairs	Stairs	Walk	Stairs	STS	Stairs	Walk	Stairs	STS	Walk	Stairs	Stairs
S10	STS	Stairs	Stairs	STS	STS	Walk	Walk	Stairs	STS	Walk	Stairs	Walk	STS	Walk	Walk	Stairs
	STS	Stairs	Stairs	STS	STS	Walk	Walk	Stairs	STS	Stairs	Stairs	STS	STS	Stairs	Walk	Walk
S11	STS	Walk	Walk	STS	Stairs	Stairs	Stairs	STS	STS	Stairs	Stairs	STS	STS	Stairs	Walk	Walk
	STS	Walk	Stairs	Stairs	Stairs	Stairs	Stairs	STS	STS	Stairs	Walk	Stairs	STS	Walk	Stairs	Stairs
S12	STS	Stairs	Walk	Stairs	STS	Walk	Walk	Walk	STS	Walk	Stairs	Stairs	STS	Walk	Walk	Stairs
	–	–	–	–	–	–	–	–	–	–	–	–	–	–	–	–
S13	STS	Walk	Walk	STS	STS	STS	STS	STS	STS	Stairs	Walk	Stairs	STS	Stairs	Walk	STS
	STS	Stairs	Stairs	Stairs	STS	Stairs	Stairs	STS	STS	Stairs	Stairs	Stairs	STS	Walk	Walk	STS
S14	STS	Stairs	Stairs	STS	Stairs	Stairs	Walk	Walk	STS	Stairs	Stairs	STS	STS	Stairs	Walk	Stairs
	STS	Stairs	Stairs	Stairs	STS	Stairs	Walk	Stairs	STS	Stairs	Stairs	STS	Stairs	Stairs	Walk	Stairs
S15	STS	Stairs	Stairs	Stairs	Stairs	Walk	Walk	Walk	STS	Stairs	Stairs	STS	Stairs	STS	Walk	Stairs
	STS	Stairs	Stairs	Stairs	Stairs	Walk	Walk	NA	STS	Walk	Stairs	Stairs	Stairs	Walk	Walk	NA
S16	Stairs	Stairs	Stairs	Stairs	Stairs	Stairs	Walk	Stairs	Stairs	Stairs	Walk	Stairs	Stairs	Stairs	Walk	Stairs
	Walk	Stairs	Stairs	Stairs	STS	Walk	Walk	Stairs	STS	Stairs	Walk	Stairs	Stairs	Walk	Walk	Stairs
S17	STS	Stairs	Walk	Stairs	Stairs	Stairs	Walk	STS	STS	Walk	Stairs	Stairs	Stairs	Stairs	Walk	STS
	STS	Stairs	Stairs	Stairs	Stairs	Stairs	Stairs	STS	STS	Stairs	Walk	Stairs	Stairs	Stairs	Walk	STS
S18	STS	Walk	Stairs	Stairs	Stairs	Walk	Stairs	Stairs	STS	Stairs	Stairs	STS	Stairs	Walk	Walk	Stairs
	STS	Walk	Stairs	Stairs	STS	Walk	Walk	STS	STS	Stairs	Stairs	STS	Stairs	Stairs	Walk	STS
S19	Walk	Stairs	Walk	Stairs	Stairs	Walk	Walk	Walk	Stairs	Stairs	Stairs	Stairs	Stairs	Walk	Walk	Stairs
	Stairs	Stairs	Walk	Stairs	Stairs	Walk	Walk	Walk	STS	Stairs	Stairs	Stairs	Stairs	Walk	Walk	Stairs
S20	STS	Stairs	Stairs	STS	STS	Stairs	Walk	STS	STS	Stairs	Stairs	STS	STS	Walk	Walk	Stairs
	STS	Stairs	Walk	Stairs	Stairs	Stairs	Stairs	STS	STS	Stairs	Stairs	STS	STS	Stairs	STS	Stairs
S21	STS	Walk	Walk	STS	STS	Walk	Walk	STS	STS	Stairs	Stairs	Stairs	STS	Walk	Walk	Stairs
	STS	Walk	Stairs	STS	STS	Stairs	Walk	Stairs	STS	Stairs	Stairs	STS	STS	Walk	Walk	STS

Kinematics and kinetics data issues/quality

An overview of issues in kinematics and kinetics data encountered during data collection and export. The issues are organised per person per session. Where no issues were detected, a dash (--) is used. Subjects S7 and S12 did not go through Session3, which is why NA (not applicable) is used. **PS** = preferred walk start; **PE** = preferred walk end; **Shortened tests** = tests with cycles removed due to overstepping; **GRF LM issues** = tests where some or all cycles had excessive noise in LM direction, making those cycles' force unusable (see Table S4 for the problematic cycles); **GRF LM/V offset** = tests where force plates had an offset that was corrected in post-processing.

Table S3. An overview of issues in kinematics and kinetics data encountered during data collection and export.

	Session 2	Session 3
**S1**	**Shortened tests**: PS, T14, T17, T20, PE; **T12**: Cycles 95 and 98 are off in joint angles on the Right side	**Shortened tests**: T1, T3; **T27**: 4 min - 1st min missing due to late recording start
**S2**	**T22**: Cycle 175 has high peak in Right Knee angle; **T25**: SterMiddle marker covered by clothes; **T29**: Cycle 91 has high peak in Left Ankle angle	**T16**: Cycles 105 and 107 are a bit off in all Right joint angles
**S3**	**Shortened tests**: PS, T6, T8, T9, T10, T17, T20, T26, T27, T28, T29, T30, PE; **T17**: Cycles 167 and 176 have spikes in Right Ankle angle; **GRF C3D at 2 kHz:** PS, T16-T20, T26-T30 (1 kHz in MAT);	**Shortened tests**: PS, T5, T11, T13, T13, T15, T25; **T24**: 7 min in C3D, shortened to 5 min in MAT; **T21**: Cycle 37 has a spike in Left Ankle angle; **PS**: Cycles 1, 186, 187, and 207 have spikes in Right Ankle angle; **GRF LM issues**: PS
**S4**	**Shortened tests**: T4, T24; **T4**: Cycles 261 & 262 off in all Left joints; **GRF C3D at 2 kHz**: PS (1 kHz in MAT)	**Shortened tests**: T12, T13, T16, T18, T20
**S5**	**Shortened tests**: PS, T1, T2, T3, T4, T5, T21, PE; **GRF LM issues**: PE, T3, T5	**Shortened tests**: PS, T6, T20, PE; **T19**: Cycle 203 has a spike in Right Knee angle; **T21**: Cycles 85, 92, 120 has NaN in Left ankle angle
**S6**	**Shortened tests**: T1, T2, T3, T4, T5, T11, T12, T13, T14, T24, PE; **GRF LM issues**: T11, T12, T13	**Shortened tests**: T20; **GRF C3D at 2 kHz**: PS
**S7**	**Shortened tests**: T2, T4, T9, T14; **T1**: Cycle 126 is off in Right Ankle angle	NA
**S8**	**Shortened tests:** PE; **All tests**: swapped LTO-LCAL & LLM-LMM markers in MAT (CSV unchanged); **PS**: GRF recording in C3D at 2 kHz (1 kHz in MAT)	**Shortened tests**: T28, PE; **All tests**: swapped LTO-LCAL and LLM-LMM markers in MAT (CSV unchanged)
**S9**	**Shortened tests**: PS, T2, T4, T5, T11, T12, T13, T14, T15, T24; **T5**: Cycle 18 has a spike in Right Ankle angle; **All tests** (excl. T11): swapped LTO-LCAL markers in MAT (CSV unchanged); **GRF LM issues**: T1, T2, T4, T5, T12, T13, T14, T15; **GRF LM offset**: T1, T2, T3, T4, T12, T21, T22, T23, T24, T25, PE; **GRF V offset**: all tests (40N); **GRF C3D at 2 kHz:** PS, PE (corrected to 1 kHz in MAT)	**Shortened tests**: T6, T9, T10, T17, T26, T27, T28, T29, T30, PE; **All tests**: swapped LTO-LCAL markers in MAT (CSV unchanged); **GRF LM issues**: T6, T7, T8, T9, T10, T26, T27, T28, T29; **GRF V offset:** all tests (40N)
**S10**	**Shortened tests**: PS, T1, T2, T3, T4, T5, T21, T23, T25, T26, T27, T28, PE; **GRF C3D at 2 kHz**: T5 (corrected to 1 kHz in MAT); **GRF V offset**: all tests (40N); **T21**: Cycle 7 spikes in Left Ankle joint; **GRF LM issues**: PS, T2, T3, T4, T5, T23, T24, T26, PE	**Shortened tests**: PS, T6, T7, T8, T9, T10, T11, T12, T13, T14, T15, T18, T19, T20, PE; **GRF V offset**: all tests (40N); **GRF LM issues**: T6, T7, T8, T9, T10, T11, T12, T13, T14 T18, T19; **T7**: Cycle 147 off in all three Left joint angles; **GRF LM offset**: PE
**S11**	**Shortened tests**: T6, T9, T10; **GRF C3D at 2 kHz:** PS, T1, T2, T3, T4, T5, T7, T9, T10, T26, T27, T28, T29, T30, PE (1 kHz in MAT); **T2**: LMM fell off after test, put back w/o re-calibration	**Shortened tests**: T12; **T12**: RLM fell off after test, put back w/o re-calibration; **T25**: swapped LTO-LCAL markers in MAT (CSV unchanged)
**S12**	**Shortened tests**: T24; **GRF C3D at 2 kHz:** T2, T5, T12; **T2, T3**: many oversteps so sub-optimal segemntation; **PE**: missing test; **T4, T5**: missing segmented data	NA

	Session 2	Session 3
**S13**	**Shortened tests:** T1, T2, T4, T5, PE	**Shortened tests**: T11, T12, T15; **Other**: MidSterDown marker missing
**S14**	**Shortened tests:** T6, T7, T8, T9, T10, T13, T15, T18; **GRF LM issues:** T11	**Shortened tests:** T4, T5; **T4**: Cycle 1 spikes in all Right joint angles; **T5**: Cycles 13, 24, 178 spike in all Right joint angles
**S15**	**Shortened tests**: PS, T7, T8, T9, T14, T15, T19, T20, PE	–
**S16**	**Shortened tests**: PS, T4, T5, T6, T7, T8, T9, PE; **GRF LM issues**: T1, T2, T3, T5, T8, PE; **T5**: two files in C3D (1st cut off at ~130 sec; 2nd starting from ~57 sec); **T7**: 2min:15sec only; **T8**: two files in C3D (1st cut off at ~80 sec; 2nd starting from ~5 sec; **T1, T2, T5**: Right hip angle not smooth in 20-40% gait across multiple cycles	**Shortened tests**: T16, T19; **GRF LM issues**: T12, T13, T21, T23, PE
**S17**	**Shortened tests**: PS, T12, T25; **PS**: Cycle 173 all Right joint angles time-shifted; **T24**: Cycles 11, 54, 74, 149, 150, 188, 198 spike on all Right joint angles; Cycles 53, 187 have spikes on Right hip angle	**Shortened tests**: T2, T4, T7, T9; **PS**: Cycles 131 and 132 all Right joint angles time-shifted
**S18**	**Shortened tests**: PS, T18, T19, PE; **GRF LM issues**: T11	**Shortened tests**: PS, T2, T5, T6, T8, T9, T27, PE; **GRF LM issues**: T5
**S19**	**Shortened tests**: T7, T16, T19; **T6**: 4 min 45 sec (data omitted from the start to fix a sensor); **T17**: swapped RLM-RMM markers in MAT (CSV unchanged)	**Shortened tests**: T5, T23; **T24**: swapped RLM-RMM markers in MAT (CSV unchanged)
**S20**	**Shortened tests**: PS, T7, T9, T10, T16, T20, T27	**Shortened tests**: PS, T5, T15; **GRF LM issues**: T13
**S21**	**Shortened tests**: T28	**Shortened tests**: T11, T19; **PS**: swapped markers RMM-RLM in MAT (CSV unchanged); **T11**: Cycles 88 and 89 are off in all Left joint angles; **T19**: RMM reconstructed from ~137 sec onwards using rigid body assumption

Ground reaction force issues

Due to high noise in some of the ground reaction force (GRF) measurements, there are tests that have partially or even completely unusable GRF signal in lateral-medial (LM) directions. Table S4 below gives an overview of subjects with unusable GRF LM signal, including which cycles have poor quality data. Across all tests and subjects, excluding S9 and S10, the minimum number of cycles (i.e., 'common denominator') is 60. S9 has several tests where GRF LM is completely useless (T5, T8, T9, T10, T14, T26); excluding these tests, the minimum number of cycles across all tests in S9 is 43. Similarly, S10 also has several tests where GRF LM is completely useless (T4, T5, T8, T10, T12); excluding these tests, the minimum number of cycles across all tests in S10 is 10.

Table S4. An overview of tests with poor quality ground reaction force in lateral-medial direction. Indicated cycles need to be removed from the analysis that involves GRF data and its derivatives (e.g., centre of mass, CoM, calculated using double integration method).

	Tests with poor quality GRF LM (cycles to be removed from analysis)	Good/total cycles
**S3**	**PS (Sess3)**: 1, 4, 11, 21, 49, 77, 88, 180, 186, 187, 197, 207, 296	**PS (Ses3):** 309/322
**S5**	**T3**: 41-169, 251-280; **T5**: 1-36, 66-99, 146-174, 196-274, 321-228; **PE (Ses2)**: 127-174, 210-371	**T3**: 121/280; **T5**: 142/338; **PE Ses2**: 161/371
**S6**	**T11:** 60, 63-85; **T12:** 63-100; **T13:** 1-27	**T11:** 61/85; **T12:** 62/100; **T13:** 93/120
**S9**	**T1:** 1-7; **T2:** 7-17, 72-74, 106-114; **T4:** 201-256; **T5, T8-T10:** remove all; **T6**: 1-15, 31-72, 99-151; **T7:** 144-196; **T12:** 112-127; **T13:** 38-40, 47-131; **T14:** remove all **T15:** 139-158; **T26:** remove all; **T27:** 75-138; **T28:** 100-141; **T29:** 43-45, 50, 51, 56-122, 127, 128, 133-135, 140, 141, 150-159	**T1:** 216/223; **T2:** 206/229; **T4:** 200/256; **T5:** 0/290; **T6:** 70/180; **T7:** 143/196; **T8;** 0/203; **T9:** 0/204; **T10:** 0/237; **T12:** 111/127; **T13:** 43/131; **T14:** 0/148; **T15:** 138/158; **T26:** 0/130; **T27:** 74/138; **T28:** 99/141; **T29:** 70/159
**S10**	**PS (Ses2):** 10, 17, 26, 35, 96, 124, 131, 163, 169, 187; **PS (Ses3):** 36-79, 123-293; **T2:** 1-3, 24-219; **T3:** 129-228; **T4, T5, T8, T10, T12:** remove all; **T6:** 129-180; **T9:** 99-216; **T11:** 41-132; **T13:** 61-146 ; **T14:** 1-94; **T18:** 22, 138, 154, 157, 162; **T19:** 71, 151, 206; **T20**: 1-48, 74-125, 163-214, 292, 292; **T23:** 191; **T24:** 48, 52, 192; **T26:** 92-163; **PE (Ses2):** 64, 68, 70, 74, 85-175, 205-308; **PE (Ses3):** 1, 2, 90-93, 166, 231-306	**PS (Ses2):** 286/296; **PS (Ses3):** 77/293; **T2:** 19/219; **T3:** 128/228; **T4:** 0/242; **T5:** 0/275; **T7:** 128/158; **T8:** 0/161; **T9:** 98/216; **T10:** 10/242; **T11:** 41/132; **T12:** remove all; **T13:** 61/146; **T14:** 94/159; **T18:** 244/249; **T19:** 296/272; **T20:** 235/292; **T23:** 222/223; **T24:** 256/259; **T26:** 91/163; **PE (Ses2):** 108/308; **PE (Ses3):** 221/306
**S14**	**T11:** 10-27, 91-125	**T11:** 72/125
**S16**	**T1:** 71-84, 241-262; **T2:** 201-285; **T3:** 121-292; **T5:** 76-144, 181-270; **T8:** 46-74; **T12:** 96-183; **T13:** 61-193; **T21:** 204-227; **T23:** 231-244; **PE (**Ses2**):** 161-244, 351-370; **PE (Ses3):** 242-282	**T1:** 226/262; **T2:** 200/285; **T3:** 120/292; **T5:** 111/270; **T8:** 148/177; **T12:** 95/183; **T13:** 60/193; **T21:** 203/227; **T23:** 230/244; **PE (Ses2):** 266/370; **PE (Ses3):** 240/382
**S18**	**T5:** 30, 66-97, 114-174, 217; **T11:** 6, 111-123	**T5:** 108/283; **T11:** 109/123
**S20**	**T13:** 61-115, 120, 121, 126-134	**T13:** 80/146
